# Enhancing WNT Signaling Restores Cortical Neuronal Spine Maturation and Synaptogenesis in *Tbr1* Mutants

**DOI:** 10.1016/j.celrep.2020.03.059

**Published:** 2020-04-14

**Authors:** Siavash Fazel Darbandi, Sarah E. Robinson Schwartz, Emily Ling-Lin Pai, Amanda Everitt, Marc L. Turner, Benjamin N.R. Cheyette, A. Jeremy Willsey, Matthew W. State, Vikaas S. Sohal, John L.R. Rubenstein

**Affiliations:** 1 Department of Psychiatry and UCSF Weill Institute for Neurosciences, University of California, San Francisco, San Francisco, CA 94143, USA; 2 Institute for Neurodegenerative Diseases, UCSF Weill Institute for Neurosciences, University of California, San Francisco, San Francisco, CA 94143, USA; 3 Kavli Institute for Fundamental Neuroscience and Sloan-Swartz Center for Theoretical Neurobiology, University of California, San Francisco, San Francisco, CA 94143, USA; 4 Quantitative Biosciences Institute (QBI), University of California, San Francisco, San Francisco, CA 94143, USA; 5 Senior author; 6 Lead Contact

## Abstract

*Tbr1* is a high-confidence autism spectrum disorder (ASD) gene encoding a transcription factor with distinct pre- and postnatal functions. Postnatally, *Tbr1* conditional knockout (CKO) mutants and constitutive heterozygotes have immature dendritic spines and reduced synaptic density. *Tbr1* regulates expression of several genes that underlie synaptic defects, including a kinesin (*Kif1a*) and a WNT-signaling ligand (*Wnt7b*). Furthermore, *Tbr1* mutant corticothalamic neurons have reduced thalamic axonal arborization. LiCl and a GSK3β inhibitor, two WNT-signaling agonists, robustly rescue the dendritic spines and the synaptic and axonal defects, suggesting that this could have relevance for therapeutic approaches in some forms of ASD.

## In Brief

Fazel Darbandi et al. demonstrate that TBR1 directly regulates transcriptional circuits in cortical layers 5 and 6, which promote dendritic spine and synaptic density. Enhancing WNT signaling rescues dendritic spine maturation and synaptogenesis defects in *Tbr1* mutants. These results provide insights into mechanisms that underlie ASD pathophysiology.

## Graphical Abstract

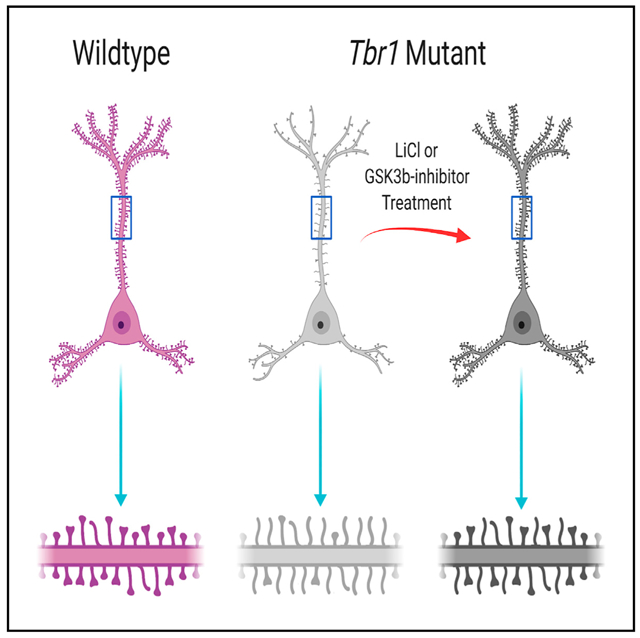

## INTRODUCTION

Autism spectrum disorders (ASDs) are defined by deficits in social interaction and abnormalities in language development and repetitive behavior. Considerable genetic and phenotypic heterogeneity has complicated efforts to understand the underlying biology of ASD. However, recent progress in the genomics of ASD has revealed more than 65 high-confidence ASD (hcASD) risk genes (Sanders et al., 2015). Systems analyses suggest that expression of ASD risk genes have important functions in mid-fetal deep-layer cortical excitatory neurons and that disruption may contribute to ASD pathophysiology (Willsey et al., 2013). Among these ASD genes, analysis of the *Tbr1* transcription factor (TF) is attractive, as it opens the possibility of defining a transcriptional pathway that includes other ASD genes.

*Tbr1* has a central role in the development of mouse early-born excitatory cortical neurons. *Tbr1* expression, which begins in newborn neurons, dictates layer 6 identity (Bedogni et al., 2010; Bulfone et al., 1998; Hevner et al., 2001, 2003; McKenna et al., 2011). Using *Tbr1^layer6^* conditional knockouts (CKOs), we recently demonstrated that neonatal *Tbr1* function in layer 6 is required for maintaining corticothalamic identity and synaptogenesis (Fazel Darbandi et al., 2018).

Here, we delved deeper into *Tbr1*’s function in synaptogenesis in several ways. First, we identified convergent synaptic pheno-types in *Tbr1^layer5^* and *Tbr1^layer6^* CKOs and *Tbr1^constitutive^* (*Tbr1*^+/−^) mutants, including a defect in the formation of mature dendritic spines. Next, we used single-cell RNA sequencing (scRNA-seq) of *Tbr1^layer5^* mutant medial prefrontal cortex (mPFC) neurons and identified *Tbr1*-regulated genes that impact synapse formation in layer 5, including a kinesin motor protein (*Kif1a*) and genes in the WNT-signaling pathway (*Gsk3β*, *Ctnnb1*, and *Wnt7b*). We also identified a number of *Tbr1*-regulated ASD genes in the layer 5 neurons of the mPFC, including *Ank2*, *Ap2s1*, *Ctnnb1*, *Dpysl2*, *Map1a*, *Rorb*, *Smarcc2*, and *Gsk3β*. Finally, we found that LiCl, a drug approved by the US Food and Drug Administration, and a GSK3β inhibitor (SB216763; Sigma-Aldrich) that promotes WNT signaling rescue the spine and synaptic defects in adult *Tbr1^layer5^*, *Tbr1^layer6^*, and *Tbr1^constitutive^* (*Tbr1*^+/−^) mutants. Lastly, *Tbr1^layer5^* mutants exhibit decreased social interactions with young mice, a phenotype that is rescued with LiCl treatment. The LiCl results suggest an important and novel biological mechanism underlying ASD that may have implications for the treatment of patients with *TBR1* mutations and, potentially, other individuals with ASD or related neurodevelopmental disorders.

## RESULTS

### *Tbr1* Regulates Genes Involved in Cytoskeletal Dynamics and Synaptogenesis in Layer 5 Pyramidal Neurons of Neonatal mPFC

In the frontal and motor cortex, *Tbr1* is expressed in most excitatory neurons in layers 5 and 6, whereas layer 5 expression in other cortical regions is limited to a minority of neurons (Bulfone et al., 1995). Here, using a floxed allele, we selectively eliminated *Tbr1* in cortical layer 5 pyramidal neurons around postnatal day (P)0 using *Rbp4-cre* ~8 days after *Tbr1* expression begins. We refer to these mice as *Tbr1^layer5^* CKOs.

**Figure 1.**
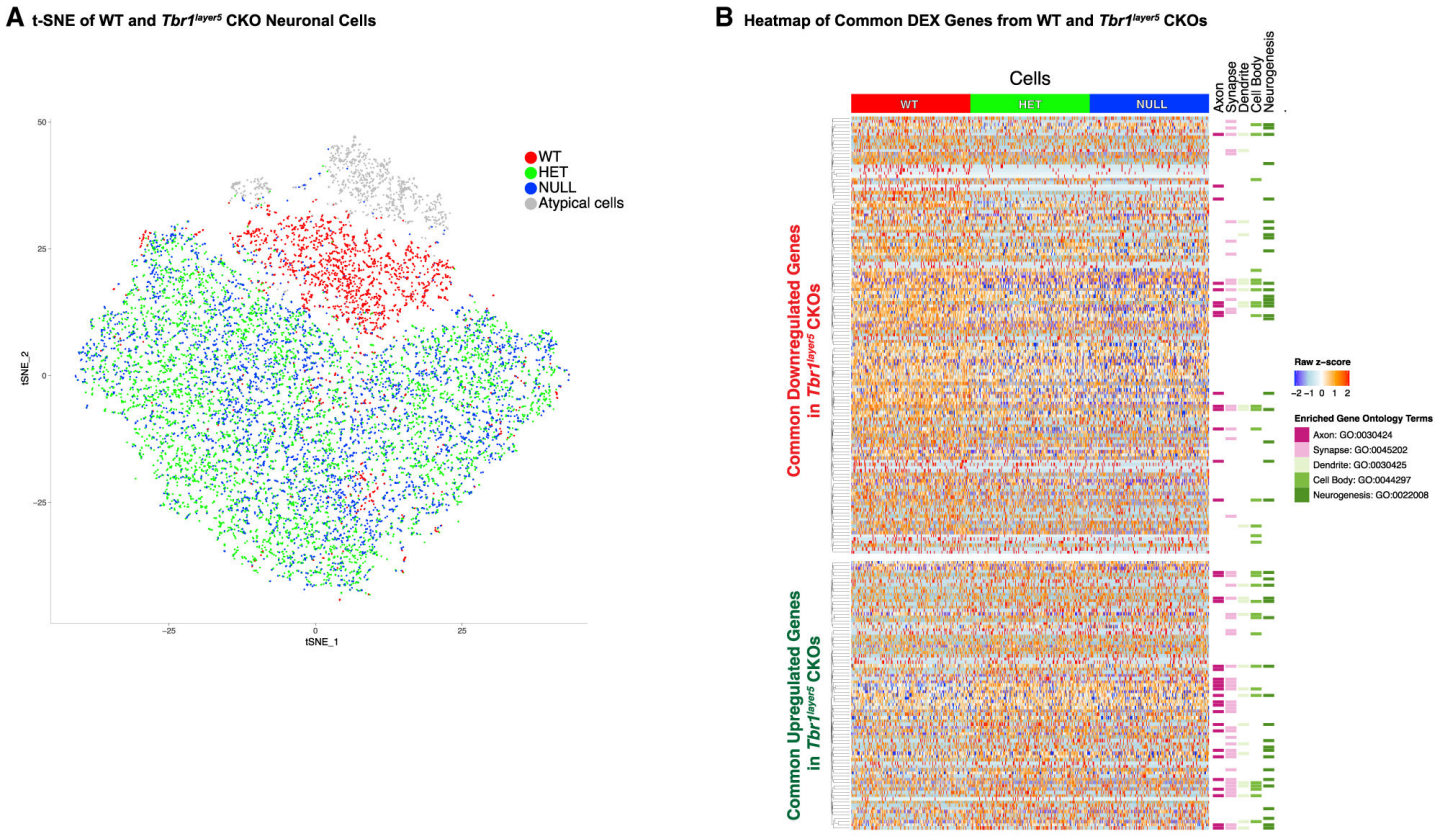
*Tbr1* Regulates Genes that Are Implicated in Controlling the Development of Axons, Synapses, and Dendrites in Layer 5 Pyramidal Neurons of the mPFC (A) *t*-distributed stochastic neighbor embedding (*t*-SNE) plot displaying 11,070 single neuronal cells from *Tbr1*^*layer5*^ WT (red) and from *Tbr1^layer5^* heterozygous (HET; green), and *Tbr1^layer5^* homozygous (NULL; blue) CKOs. t-SNE was performed after quality control and removal of non-neuronal cell subtypes. (B) Heatmap of DEX genes (FDR < 0.05) shared between both genotypes (x axis, n = 218) over a randomly selected 1,000 cells from each genotype (y axis, n = 3,000). Genes are ordered by hierarchal clustering within direction of regulation grouping, and the *Z* score of normalized gene expression data is shown. The genotype for each cell is depicted at the top, and genes with membership in selected enriched GO categories are highlighted at the right. See also Figures S1-S3.

We focused on *Tbr1* function in the developing prefrontal cortex (PFC), a region that is implicated in ASD (Willsey et al., 2013). To overcome the limitations caused by cellular heterogeneity of batch RNA sequencing (RNA-seq) (*Tbr1* is expressed in ~60% of layer 5 pyramidal neurons at P5 and ~85% at P21; Figures S1A and S1B), we generated scRNA-seq data from fluorescence-activated cell sorting (FACS) of layer 5 neurons isolated from P5 mPFC (Figure 1). We studied the transcriptomic changes from *Tbr1*^*wild-type*^, *Tbr1^layer5^* heterozygous and homozygous CKO cells using the 10X Genomics platform (GenBank GEO: GSE146298).

To identify genotype-dependent gene expression changes, we used a t-distributed stochastic neighbor embedding (t-SNE) dimensionality reduction followed by differential expression (DEX) analysis to identify neuronal cells (Figures 1A and S2). We captured 11,070 cells and 7,174 genes from *Tbr1^wild-type^* (n = 1,778 cells), *Tbr1^layer5^* heterozygous (n = 5,357 cells), and *Tbr1^layer5^* homozygous (n = 3,935 cells) mutant mPFCs that were used for downstream analysis (Figure S2). We excluded 873 cells classified as atypical neuronal cells, with lower expression levels of *Neurod6* and *Nrgn* (two excitatory neuronal markers; Figures S3A and S3D) and high levels of housekeeping genes, from DEX analyses (gray cells in Figure 1A). The t-SNE plot demonstrated clear separation between *Tbr1^wid-type^* and *Tbr1^layer5^* CKOs (Figure 1A). DEX analysis identified 470 DEX genes when comparing *Tbr1^layer5^* homozygous mutants to *Tbr1*^*wild-type*^ (Table S1) and 320 DEX genes when comparing *Tbr1ayer5* heterozygous mutants to *Tbr1^wild-type^* (Table S2), 218 of which occur in both comparisons (false discovery rate [FDR] ≨ 0.05) (Figure 1B, Table S3). Feature plots showing the expression of layer 5 markers in our scRNA-seq cell population are shown (Figure S3i). Gene Ontology (GO) analysis of DEX genes identified terms including “axon,” “synapse,” “dendrite,” “cell body,” and “neurogenesis” (Figure 1B; Table S4).

To determine whether the changes in gene expression in *Tbr1^layer5^* CKOs are due to direct regulation by TBR1, we used data from TBR1 chromatin immunoprecipitation (ChIP-seq) from P2 wild-type (WT) cortex (Fazel Darbandi et al., 2018). TBR1 binds to the promoters and distal regions of layer 5 DEX genes (within 100 kb) (Figure S1C). This suggests that TBR1 may be involved in controlling the expression by activating or repressing the target genes.

**Figure 2.**
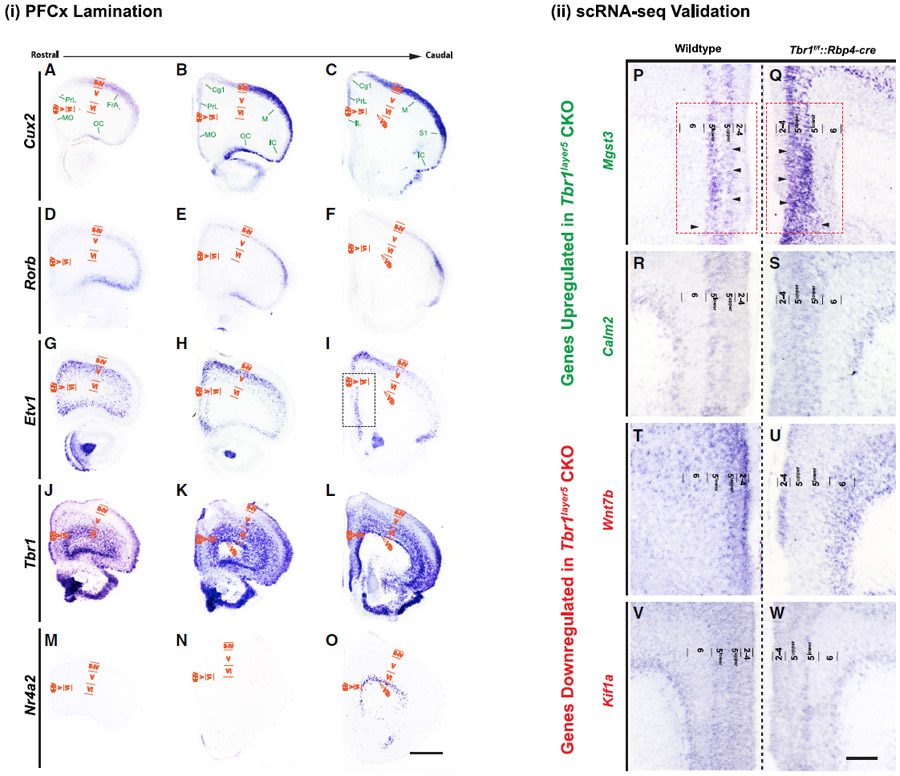
*Tbr1* Regulates Expression in the mPFC *In situ* hybridization defines rostral cortical lamination and validates the changes in scRNA-seq expression levels. (i) PFC lamination. Prefrontal cortical lamination was defined using ISH on coronal sections of neonatal mPFC in WT mice at P3. (A–O) ISH was performed on rostral, medial, and caudal areas, respectively, using (A–C) *Cux2* (layers 2–4); (D–F) *Rorb* (layer 4); (G–I) *Etv1* (layer 5); (J–L) *Tbr1* (layers2/3, 5, and 6); and (M–O) *Nr4a2* (subplate or layer 6b). Cortical layers in the medial and dorsal regions are labeled. MO, medial orbital cortex; PrL, prelimbic cortex; FrA, frontal association cortex; OC, orbital cortex; Cg1, cingulate cortex area 1; M, motor cortex; S1, primary somatosensory cortex; IC, insular cortex; II-IV, layers 2–4; V, layer 5; VI, layer 6; VIb, subplate. Scale bar, 300 μm. (ii) scRNA-seq validation. ISH confirms the changes in the transcriptome changes from DEX analysis of scRNA-seq in *Tbr1^layer5^* homozygous mutants. (P–W) The expression of *Mgst3* (P and Q) and *Calm2* (R and S) are increased in layer 5^upper^ (Q and S). *Tbr1^layer5^* mutants exhibit reduced expression of *Wnt7b* (T and U) and *Kif1a* (V and W) in layer 5 of the mPFC at P3. Only one hemisphere is shown from the ISH images from WT and *Tbr1^layer5^* homozygous CKOs, which are presented as mirror images, to aid in evaluating the changes in laminar gene expression. Color code: downregulated (red) and upregulated (green). Red box shown in (P) and (Q) indicates the region that was dissected for scRNA-seq analyses. Cortical layers 2–4, 5^upper^, 5^lower^, 6, and 6b (subplate) are labeled. Scale bar, 100 μm. See also Figure S3.

We used *in situ* hybridization (ISH) to validate the expression of several DEX genes (Table S5). Our scRNA-seq analysis in conjunction with ISH aided in discovering *Mgst3*, as a new layer 5 marker of prefrontal cortex (Figure S1D). To provide a histological context, we defined laminar boundaries in the prefrontal cortex at P3 using the following probes: *Cux2* (layers 2 and 3), *Rorb* (layer 4), *Etv1* (layer 5), *Tbr1* (layers 2–3, 5, 6, and 6b), *Nr4a2* (subplate; Figure 2i). Cortical layers 2–4 appear as a single layer at this stage (Figures 2A–2F). Expression of *Calm2*, *Kif1a*, *Mgst3*, and *Wnt7b* was altered as suggested by the scRNA-seq analysis (Figure 2ii; Figure S3ii). Thus, neonatal *Tbr1* expression in layer 5 pyramidal neurons directly regulates the expression of genes involved in cytoskeletal dynamics and synapse development.

### Excitatory and Inhibitory Synapses Are Reduced in *Tbr1^layer5^* Mutants

**Figure 3.**
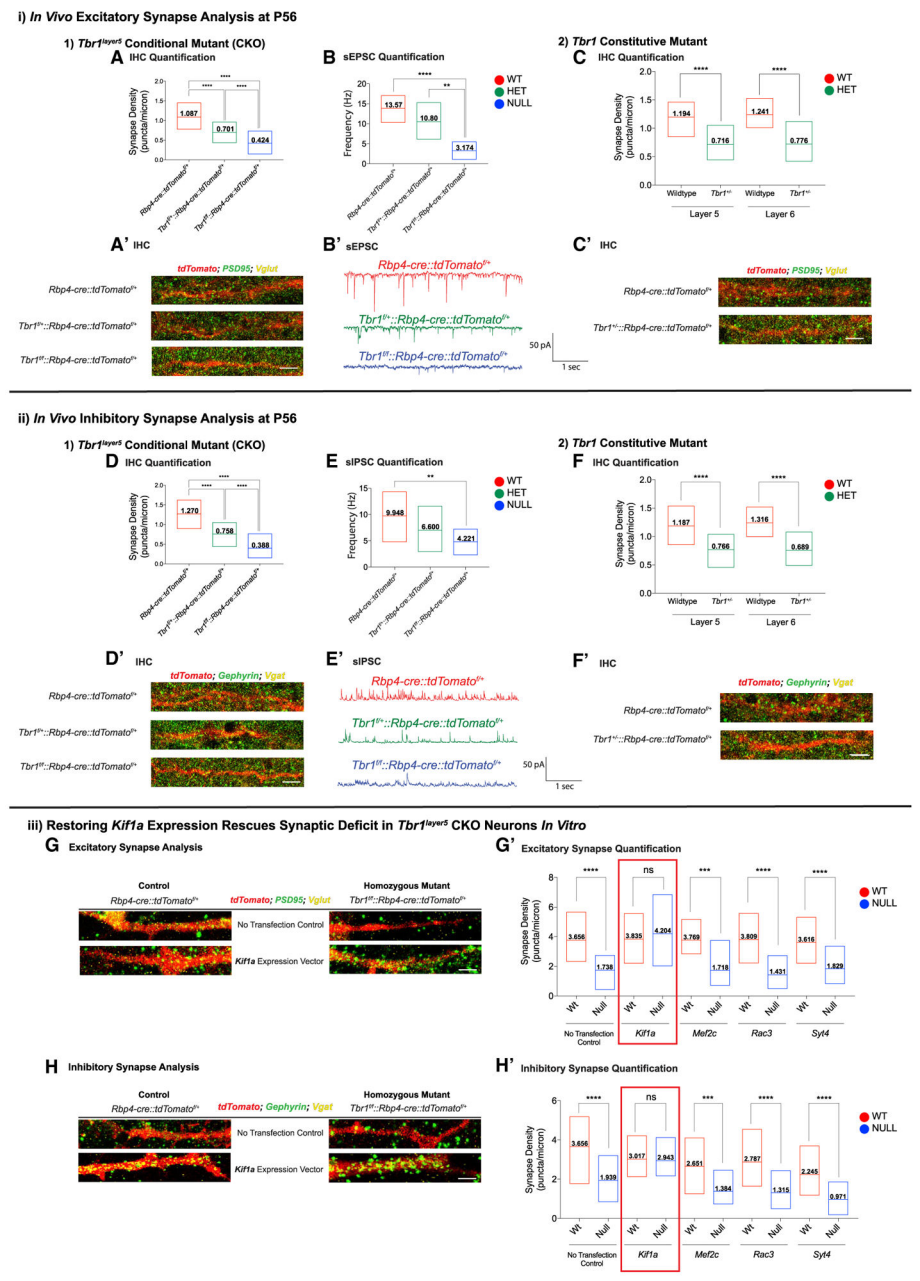
*Tbr1* Mutants Have Reduced Excitatory and Inhibitory Synaptic Densities at P56 IF was used to detect excitatory (i) and inhibitory (ii) synapses onto dendrites of (1) the mPFC of *Tbr1^wild-type^* (*Rbp4-cre*∷*tdTomato*^*f*/+^; red), *Tbr1^layer5^* heterozygous (*Tbr1*^*f*/+^∷**Rbp4-cre**∷*tdTomato*^*f*/+^; green), and *Tbr1^layer5^* homozygous (*Tbr1^f/f^*∷**Rbp4-cre**∷*tdTomato*^*f*/+^; blue) mutants (n = 30 dendrites), and (2) dendrites of layer 5 neurons from the mPFC of *Tbr1^wild-type^*, *Tbr1*^+/−^ and layer 6 neurons from the SSCx of *Tbr1^wild-type^*, *Tbr1*^+/−^ (n = 15 dendrites). (A–C′) Excitatory synapses were identified by colocalization of VGLUT1^+^ boutons and PSD95^+^ clusters on dendrites of layer 5 pyramidal neurons at P56 (A′ and C′). (A) Quantification of excitatory synaptic density. (B) Quantification of the sEPSC frequency from layer 5 neurons at P56 (n = 6/6/6, WT/heterozygous/homozygous cells from two different animals per genotype). (B′) Sample traces of sEPSC recordings at P56. (C) Quantification of excitatory synaptic density of *Tbr1*^+/−^ mutants in cortical layers 5 and 6 at P56. (D–F′) Inhibitory synapses were identified by co-localizing VGAT^+^ boutons and Gephyrin^+^ clusters (D′ and F′). (D) Quantification of inhibitory synaptic density on dendrites of layer 5 pyramidal neurons at P56. (E) Quantification of the sIPSC frequency from layer 5 neurons at P56 (n = 7/7/7, WT/heterozygous/homozygous cells from two different animals per genotype). (E′) Sample traces of sIPSC recordings at P56. (F) Quantification of inhibitory synapse numbers on dendrites of layer 5 and 6 pyramidal neurons of *Tbr1*^+/−^ mutants at P56. (iii) *In vitro* rescue assay was conducted by transfecting *Kif1a*, *Mef2c*, *Rac3*, and *Syt4* expression vectors into P0 primary cortical culture from *Tbr1^wild-type^* (red) and *Tbr1^layer5^* CKOs (blue) (n = 3 biological replicates). (G–H′) Excitatory (G) and inhibitory (H) synaptic density was analyzed 14 days post-transfection. Quantification of excitatory (G′) and inhibitory (H′) synaptic density *in vitro* is indicated. Red box indicates a successful rescue of synaptic density. Two-way ANOVA was used for the statistical analysis of the control, heterozygote, and null. Two-tailed t test with Tukey correction was used for pairwise comparisons. Floating bar graphs represent the minimum-to maximum (min-max) distribution of synaptic density and/or EPSC/IPSC frequency measured from each genotype. Horizontal line in each box denotes the average distribution. Average distribution is numerically indicated in each box (**p < 0.01; ***p < 0.001; ****p < 0.0001). ns, not significant. See also Figures S4 and S5.

We assessed excitatory synapse numbers on apical dendrites of layer 5 neurons (within layer 2-3) in the mPFC by analyzing VGLUT1^+^ presynaptic terminals that are apposed to PSD95^+^ postsynaptic zones at P56 (Figure 3A′) and P21 (Figures S4D-S4F) using immunofluorescence (IF) and confocal microscopy. Inhibitory synaptic density was assessed by counting the overlapping VGAT^+^ presynaptic inhibitory terminals and Gephyrin^+^ dendritic postsynaptic zones on the apical dendrites of layer 5 pyramidal neurons (n = 30 dendrites) at P56 (Figure 3D′) and P21 (Figures S4J-S4L). Excitatory and inhibitory synapses were decreased 34% and 42% in *Tbr1^layer5^* heterozygous and 70% and 73% in *Tbr1^layer5^* homozygous mutants at P56, respectively (Figures 3A and 3D). A similar synaptic deficit was also present at P21 (Figures S4G and S4M).

To assess the physiological consequences of the decrease in excitatory and inhibitory synaptic densities, we measured spontaneous excitatory and inhibitory post-synaptic currents (sEPSCs and sIPSCs, respectively) using whole-cell patch clamp on the tdTomato^+^ layer 5 pyramidal cells in mPFC brain slices at P56 and P21 (Figures 3B′ and 3E′; Figures S4H and S4N). The sEPSC frequency was reduced 25% in *Tbr1^layer5^* heterozygous and 75% in *Tbr1^layer5^* homozygous mutants; furthermore, the frequency of sIPSCs was reduced 30% in *Tbr1^layer5^* heterozygous and 50% in *Tbr1^layer5^* homozygous mutants as compared to cells from *Tbr1^wild-type^* mice at P56 (Figures 3B and 3E). Similar decreases were also present at P21 (Figures S4I and S4O). We did not observe changes in the amplitude of sEPSCs and sIPSCs at P21 and P56 (data not shown).

Since most *de novo* ASD-risk genes are heterozygous, loss-of-function, rare variants, we explored the consequence of constitutive *Tbr1* haploinsufficiency on synapse numbers of layer 5 and layer 6 pyramidal neurons using *Tbr1*^+/−^ mice (Bulfone et al., 1995). We counted excitatory and inhibitory synapse numbers in the mPFC of *Tbr1*^+/−^∷*Rbp4-cre*∷*tdTomato*^*f*/+^ (layer 5 neurons) and the somatosensory cortex (SSCx) of *Tbr1*^+/−^∷*Ntsr1-cre*∷*tdTomato*^*f*/+^ (layer 6 neurons) at P56 (Figure 3, i2 and ii2). Layer 5 excitatory and inhibitory synapse numbers were reduced ~40% and ~35% in the mPFC of *Tbr1*^+/−^∷*Rbp4-cre*∷*tdTomato*^*f*/+^ at P56 (Figures 3C and 3F). Layer 6 neurons in the SSCx of *Tbr1*^+/−^∷*Ntsr1-cre*∷*tdTomato*^*f*/+^ showed ~37% and ~39% decreases in excitatory and inhibitory synaptic densities, respectively (Figures 3C and 3F). Thus, *Tbr1* haploinsufficiency results in reduced synaptic density on the excitatory neurons of cortical layers 5 and 6.

### *Kif1a* Expression Restores Normal Synapse Numbers in *Tbr1^layer5^* Mutant Neurons *In Vitro*

We sought to identify molecular mechanisms underlying the decrease in the excitatory and inhibitory synaptic densities in *Tbr1^layer5^* CKO neurons using the results from the scRNA-seq analysis (Figure 1). We assessed a subset of DEX genes that control synapse biology, including *Kif1a* (Li et al., 2016), *Mef2c* (Barbosa et al., 2008), *Rac3*, and *Syt4* (Barber et al., 2009). We examined whether *Kif1a*, *Mef2c*, *Rac3*, and *Syt4* could rescue synapse density by expressing them in P0 primary cortical cultures derived from *Tbr1^wild-type^* and *Tbr1^layer5^* mutant neurons (n = 3 biological replicates).

After 14 days *in vitro*, we analyzed the number of excitatory (VGLUT^+^ presynaptic and PSD95^+^ postsynaptic) and inhibitory (VGAT^+^ presynaptic and Gephyrin^+^ postsynaptic) terminals of *Tbr1^wlid-type^* and *Tbr1^layer5^* homozygous mutant neurons (Figure 3, iii). The reduced excitatory and inhibitory synaptic densities onto *Tbr1^layer5^* CKO neurons were recapitulated *in vitro* (Figures 3G, 3G′, 3H, and 3H′). Only *Kif1a* rescued the reduction in both excitatory (Figures 3G and 3G′) and inhibitory (Figures 3H and 3H′) synapse numbers. *Kif1a*, a kinesin motor protein, is implicated in the transport of vesicles for synapse development (Guedes-Dias et al., 2019) and thus may contribute to *Tbr1*’s function in promoting synapse formation.

### *Tbr1^layer5^* CKOs Have Increased Hyperpolarization-Activated Cation Currents (I_h_s)

We next examined the intrinsic properties of layer 5 neurons in *Tbr1^layer5^* WT and CKOs using whole-cell patch clamp to measure intrinsic physiological properties of *Rbp4-cre*∷*tdTomato*^+^ neurons of layer 5 in the mPFC (Figure S5A). Resting membrane potential and input resistance were not different between *Tbr1^wild-type^*, *Tbr1^layer5^* heterozygotes and homozygotes at P56 (Figures S5B and S5C).

A prominent feature of many layer 5 pyramidal neurons is the presence of an I_h_ (or h-current) mediated by hyperpolarization-activated cyclic nucleotide–gated HCN channels (Shepherd, 2013). I_h_ causes a characteristic “sag” and “rebound” in current clamp recordings of responses to steps of hyperpolarizing current. We examined responses to a −200-pA step and found that mPFC layer 5 pyramidal neurons from P56 *Tbr1^layer5^* heterozygotes and homozygotes exhibited a significantly increased “sag and rebound” compared to *Tbr1^wild-type^* controls, suggesting increased I_h_, while other intrinsic electrophysiological properties were largely unaltered (Figure S5D).

In deep-layer neocortical pyramidal neurons, the presence of I_h_ shifts the resonant frequency toward higher frequencies (Dembrow et al., 2010). Therefore, to further characterize potential increases in I_h_ in *Tbr1^layer5^* CKOs, we estimated the resonant frequency by injecting constant current to hold *Rbp4-cre*^+^ neurons in current clamp near −70 mV and then introduced a sinusoidal current stimulus with constant amplitude (100 pA, peak to peak) and a frequency that increased linearly from 0 to 20 Hz over 20 s (Figure S5E). *Tbr1^layer5^* heterozygous and homozygous CKOs exhibited an increase in their resonant frequency compared to *Tbr1^wild-type^* controls at P56 (Figure S5G).

Lastly, we blocked I_h_ by bath applying the specific HCN channel antagonist ZD7288 (25 μM; Figure S5F). The resonant frequency was reduced by over 50% in the *Tbr1^layer5^* heterozygous and *Tbr1^layer5^* homozygous CKOs (Figure S5G). Thus, both *Tbr1^layer5^* heterozygotes and homozygotes have an increased I_h_ in layer 5 pyramidal neurons of the mPFC.

### *Tbr1* Mutants Have Reduced Mature Dendritic Spine Density

**Figure 4.**
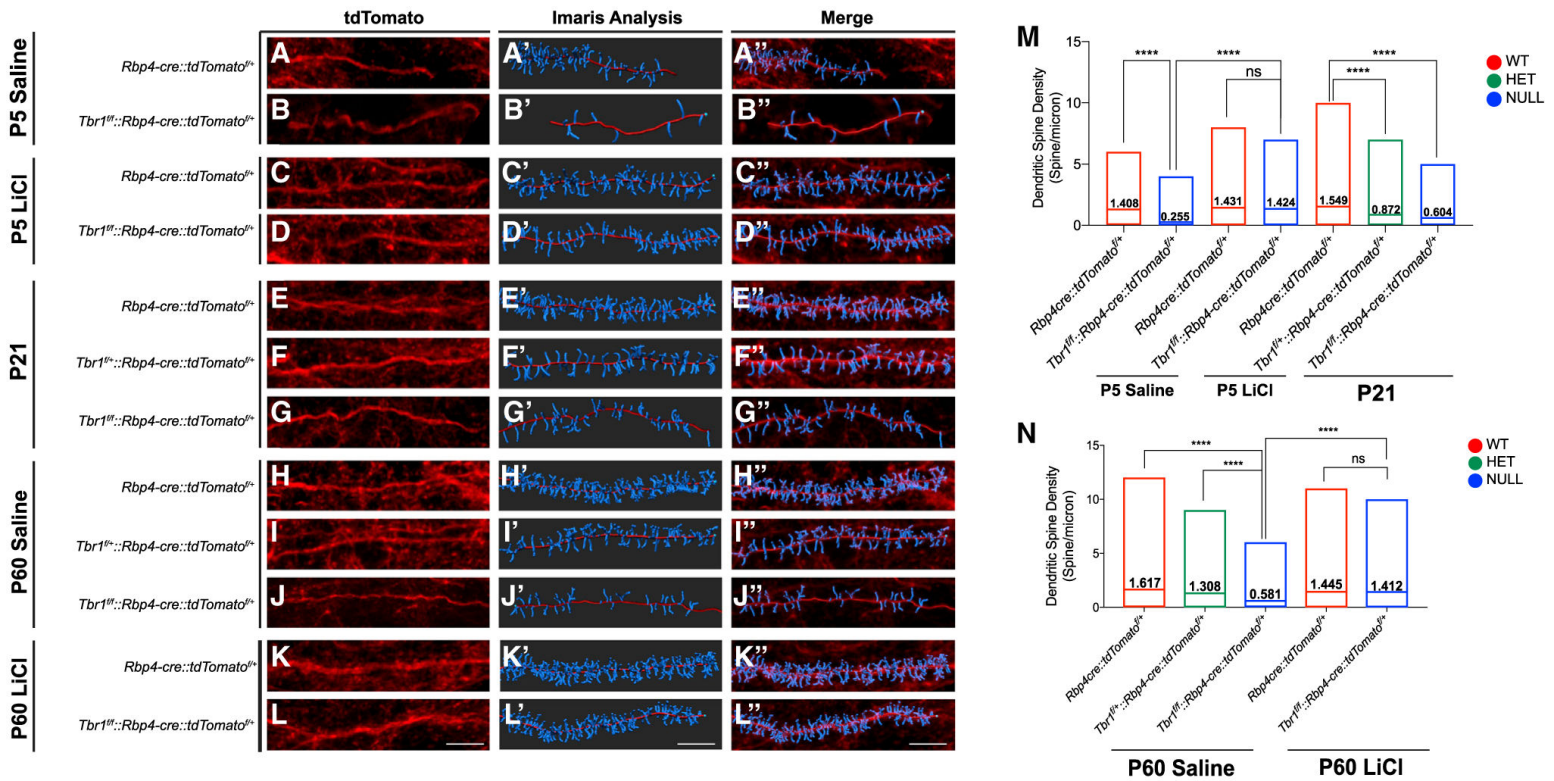
LiCl Rescues Dendritic Spine Density of *Tbr1^layer5^* CKOs (A–L″) In (A)–(L), **Rbp4-cre**∷*tdTomato*^*f*/+^ allele was used to label the dendrites of layer 5 neurons. Imaris software was used to analyze the dendritic spine density on the apical dendrites of *Tbr1^layer5^* WT and *Tbr1^layer5^* CKO neurons located within layers 2–4 of the mPFC (A′–L′). Changes in the dendritic spine density of layer 5 neurons were examined at P5 (A–D), P21 (E–G), and P60 (H–L). (A″–L″) Merged images. (M) Quantification of dendritic spine density at P5 and P21. Spine density was improved 24 hr after LiCl treatment at P5 in (C) and (D) and P60 in (K) and (L), compared to the saline-injected control animals in (A) and (B) and in (H) and (J). (N) Quantification of mature dendritic spines of *Tbr1^layer5^* WT and mutant neurons at P60, 24 h after injection with saline (control) or LiCl. Floating bar graphs represent min-max distribution of the dendritic spine density of layer 5 neurons within layers 2–4 of the mPFC. Horizontal line in each box denotes the average spine density. Average mature dendritic spine density is numerically indicated in each box. ****p < 0.0001. ns, not significant. Scale bar, 8 μm. See also Figure S6.

The synaptic deficits described earlier prompted us to investigate the state of dendritic spines in *Tbr1^layer5^* CKOs, *Tbr1^layer6^* CKOs (Fazel Darbandi et al., 2018), and *Tbr1*^+/−^ mutants. We visualized tdTomato^+^ spines using Airyscan confocal microscopy to capture 120×-magnification z stack images (using 2× optical zoom) from the dendrites of layer 6 and layer 5 neurons of WT, *Tbr1^layer5^* (Figure 4), *Tbr1^layer6^*, and *Tbr1*^+/−^ mutant neurons at P5, P21, and P60 (Figure S6). We used Imaris software (v.9.2.1) to analyze dendritic spine morphology, density, and distribution.

There were reductions in the density of mature dendritic spine density in *Tbr1* heterozygotes and homozygotes in *Tbr1^layer5^* and *Tbr1^layer6^* CKOs (Figures 4 and S6). Additionally, *Tbr1*^+/−^ mutants have reduced mature spine density on the dendrites of layer 5 and layer 6 pyramidal neurons (Figure S6). Furthermore, *Tbr1* mutant neurons had an increased filamentous spine density (Figure S6). Thus, this defect in mature dendritic spine density may underlie the reduction in synapse numbers in *Tbr1* mutants.

### Restoring Reduced WNT Signaling in *Tbr1* CKOs Rescues Synaptic Deficits

We demonstrated that *Tbr1* promotes synaptogenesis onto layer 6 neurons in part via WNT signaling through *Wnt7b* (Fazel Darbandi et al., 2018). WNT signaling promotes dendrite maturation and synapse formation (Ciani and Salinas, 2005). Here, we found several lines of evidence to further support the role of *Tbr1*-dependent WNT signaling in synapse development. First, *Wnt7b* and *Ctnnb1* expression was reduced in the mPFC of *Tbr1^layer5^* CKOs (Figures 1 and 2; Tables S1 and S2). *Ctnnb1* encodes β-catenin, the critical intracellular transducer of canonical WNT signaling (Budnik and Salinas, 2011). Second, *Tbr1^layer5^* CKOs had increased *Gsk3β* RNA expression (Figure 1); GSK3β negatively regulates WNT signaling through increasing the destruction of β-catenin (van Noort et al., 2002).

Thus, we tested whether promoting WNT signaling could rescue dendritic spine and synapse phenotypes. Among its several pharmacological effects, there is evidence that LiCl, a WNT-signaling agonist, promotes synapse development (Farooq et al., 2017; Lenox and Wang, 2003; Martin et al., 2018). Thus, we administered LiCl and a GSK3β inhibitor (SB216763, Sigma-Aldrich) to *Tbr1* mutants.

### LiCl Treatment of *Tbr1* Mutants Restores Dendritic Spine Density and Synapse Development

As noted earlier, *Tbr1* mutants have a reduced density of mature dendritic spines (Figures 4 and S6). We tested whether promoting WNT signaling by administering LiCl at P5 and P59 could rescue the reduction in mature spine density and synaptogenesis in *Tbr1* mutants. We gave a single intraperitoneal (i.p.) injection of 400 mg/kg LiCl; control animals received a single i.p. injection of 4 mL/kg saline. Impressively, LiCl treatment rescued the density of mature dendritic spines within 24 h in *Tbr1* mutants; LiCl did not have a clear effect on the density of WT dendritic spines (Figures 4 and S6). These results, in combination with the previously reported evidence that *Wnt7b* restores synapse numbers on *Tbr1^layer6^* mutant neurons (Fazel Darbandi et al., 2018), led us to test whether LiCl can rescue synapse numbers on adult *Tbr1* mutant layer 5 and layer 6 neurons.

**Figure 5.**
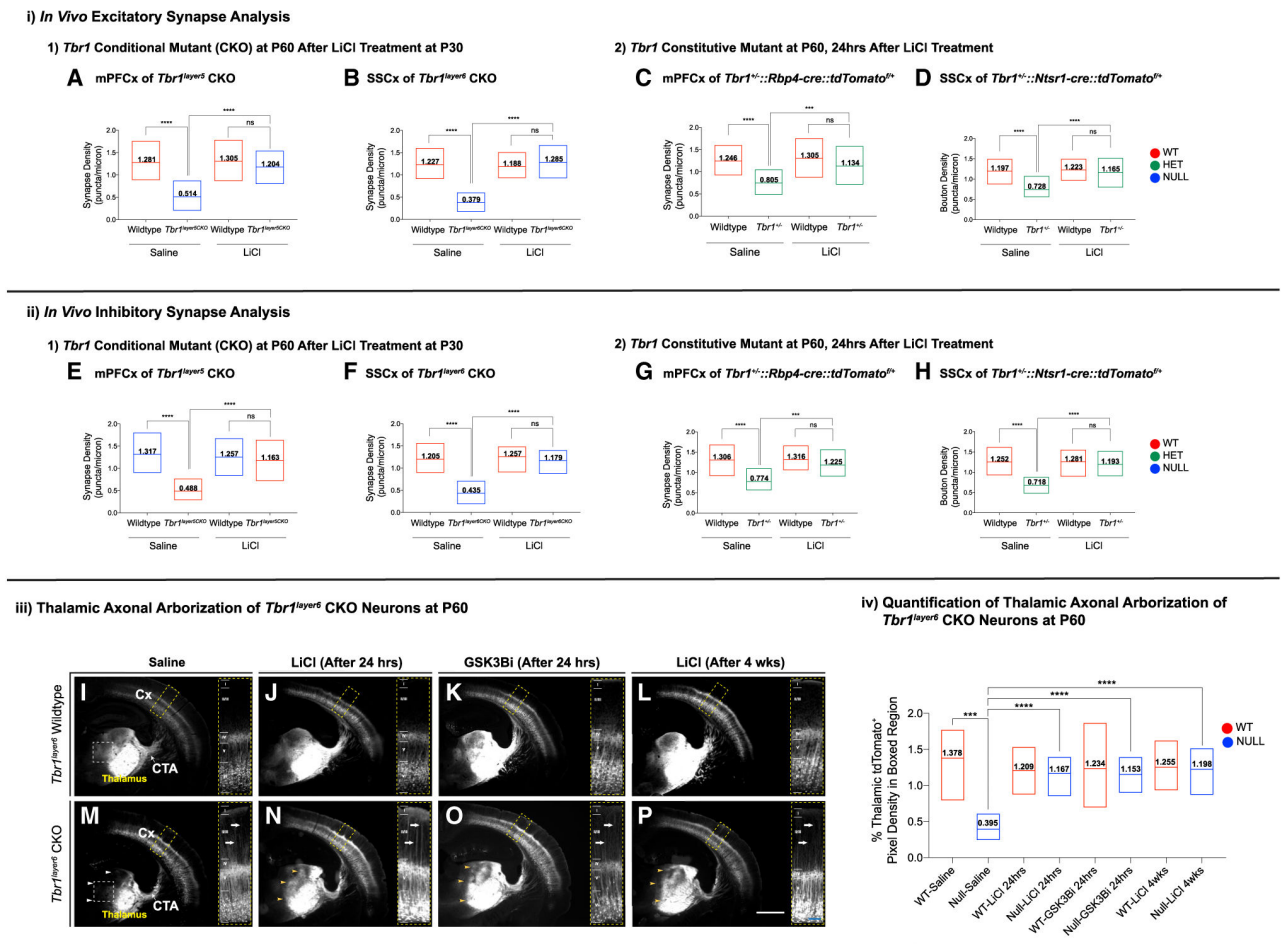
LiCl Treatment Restores Synapse Numbers and Corticothalamic Axonal Arborization of *Tbr1* Mutant Mice Excitatory (i) and inhibitory (ii) synaptic densities were quantified at P60 from: (1) apical dendrites of *Tbr1^layer5CKO^* and *Tbr1^layer6CKO^* mice 4 weeks after P30 injection with saline or LiCl (n = 15 dendrites) and (2) dendrites of layer 5 neurons from the mPFC of *Tbr1^wiid-type^*, *Tbr1*^+/−^ and layer 6 neurons from the SSCx of *Tbr1^wild-type^*, *Tbr1*^+/−^ mice 24 h after injection with saline or LiCl at P59 (n = 15 dendrites). (A and B) Excitatory synapses were quantified from (A) layer 5 neurons of the mPFC of *Tbr1^wild-type^* (green) and *Tbr1^layer5CKO^* (orange) mice and (B) layer 6 neurons from the SSCx of *Tbr1^wild-type^* (red) and *Tbr1^layer6CKO^* (blue) mice at P60, 4 weeks after saline and/or LiCl was administered. (C and D) Quantification of excitatory synaptic density of (C) layer 5 neurons of the mPFC of *Tbr1^wild-type^* (green) and *Tbr1*^+/−^ (orange) mice and (D) layer 6 neurons from the SSCx of *Tbr1^wild-type^* (red) and *Tbr1*^+/−^ (blue) mice at P60, 24 h after injection with saline or LiCl. (E and F) Inhibitory synapses were quantified from (E) the mPFC of *Tbr1^wild-type^* and *Tbr1^layer5CKO^* and (F) the SSCx of *Tbr1^wild-type^* and *Tbr1^layer6CKO^* mice 4 weeks after saline and/or LiCl was administered at P30. (G and H) Inhibitory synapses were quantified from (G) layer 5 neurons of the mPFC of *Tbr1^wild-type^* and *Tbr1*^+/−^ and (H) layer 6 neurons of the SSCx of *Tbr1^wild-type^* and *Tbr1*^+/−^ mice at P60 24 h after injection with saline or LiCl. Floating bar graphs represent the min-max distribution of all excitatory and inhibitory synapse numbers measured from each genotype. Horizontal line in each box denotes the average distribution. Average distribution is numerically indicated in each box. Two-tailed t test with Tukey correction was used for pairwise comparisons (***p < 0.001; ****p < 0.0001). ns, not significant. (I–P) In section iii, corticothalamic axonal arborization in the thalamus is indicated by tdTomato’s endogenous fluorescence of *Tbr1^layer6^* WT (I–L) and *Tbr1^layer6^* homozygous CKO (M–P) mice. The monochrome tdTomato signal (white) is indicated from saline-injected (I and M) mice, 24 h after LiCl injection (J and N), 24 h after GSK3β-inhibitor injection (K and O), and 4 weeks after LiCl injection (L and P). White arrowheads in (M) indicate thalamic regions that have reduced corticothalamic axonal arborization in *Tbr1^layer6^* CKO. Yellow arrowheads in (N)–(P) correspond to improved corticothalamic axonal arborization in *Tbr1^layer6^* CKO at P60 following LiCl treatment after 24 h (N), GSK3β-inhibitor (GSK3βi) treatment after 24 h (O), and LiCl treatment after 4 weeks (P). Yellow box depicts a high magnification of the SSCx, demonstrating that LiCl and GSK3β-inhibitor treatments did not rescue the layer 6 apical dendrite morphogenesis in *Tbr1^layer6^* CKOs. Thalamus, cortex (Cx), and corticothalamic axons (CTAs) are labeled. Scale bars: white, 1 mm; blue, 50 μm. (iv) Quantification of the tdTomato pixel intensity in the boxed regions in (I) and (M) from saline-injected *Tbr1^wild-type^* (WT-Saline) and *Tbr1^layer6^* homozygous mutants (Null-Saline) at P60. tdTomato signal intensity is improved in the thalamus of the *Tbr1^layer6^* homozygous CKO 24 h and 4 weeks after treatment compared to treatment of *Tbr1^wild-type^* at 24 h and 4 weeks. Two-tailed t test with Tukey correction was used for pairwise comparisons. Floating bar graphs represent the min-max distribution of tdTomato pixel density measured from region 1 of all genotypes and treatments. Horizontal line in each box denotes the average distribution. Average distribution is numerically indicated in each box (***p < 0.001; ****p < 0.0001). See also Figures S7, S8, and S9.

We administered LiCl to *Tbr1^layer5^* WT and homozygous CKOs (Figures 5A and 5E), *Tbr1^layer6^* WT and homozygous CKOs (Figures 5B and 5F), and *Tbr1*^+/−^ mutants (Figure 5). Layer 5 and layer 6 projection neurons were labeled with *Rbp4-cre*∷*tdTomato*^*f*/+^ and *Ntsr1-cre*∷*tdTomato*^*f*/+^, respectively. The control and LiCl-treated brains were harvested either 24 h or 4 weeks after injection at P60 (Figures 5 and S7). Confocal images of IF from the mPFC (layer 5) and SSCx (layer 6) showed a nearly complete rescue of synaptic densities, 24 h and 4 weeks after treatment (Figures 5 and S7). LiCl treatment also rescued synaptic densities in the mPFC (layer 5) and SSCx (layer 6) of the constitutive *Tbr1*^+/−^ mutants (Figure 5).

Thus, LiCl treatment of *Tbr1^layer5^*, *Tbr1^layer6^*, and *Tbr1*^+/−^ mutant mice at P60 rescues both excitatory and inhibitory synaptic deficit in *Tbr1* mutant neurons of cortical layers 5 and 6 (Figures 5A-5H). Here, we postulate that *Tbr1* mutant neurons are in a “poised” state but are not able to form synapses due to a defect in WNT signaling. Thus, we provide *in vivo* evidence that augmenting WNT signaling via LiCl treatment is sufficient to restore normal synapse numbers.

### GSK3β Inhibitor Restores Defects in Dendritic Spine and Synaptic Density of *Tbr1* Mutants

Promoting WNT signaling via LiCl treatment of *Tbr1* mutants rescued the defects in mature spine and synaptic density (Figures 5 and S7). Lithium’s best validated mechanisms of action are inhibitory effects on IMP and INPP1, central phosphatases in the phosphoinositide pathway, and on GSK3β, the central kinase in the Wnt/β-catenin and AKT pathways (Lenox and Wang, 2003). To ascertain whether WNT signaling is the main mechanism underlying the defects in dendritic spine and synaptic density of *Tbr1* mutants, we used a GSK3β inhibitor (SB216763; Sigma-Aldrich).

A single i.p. injection of GSK3β inhibitor (10 mg/kg) was given to *Tbr1* CKOs and WT at P59. Control animals received a single i.p. injection of 4 mL/kg saline at P59. We studied the effects of these treatments on *Tbr1^layer5^* WT and homozygous CKOs (Figure S8i) and on *Tbr1^layer6^* WT and homozygous CKOs (Figure S8ii). Layer 5 and layer 6 projection neurons were labeled with *Rbp4-cre*∷*tdTomato*^*f*/+^ and *Ntsr1-cre*∷*tdTomato*^*f*/+^, respectively. The control and GSK3β-inhibitor-treated brains were harvested after 24 h (Figure S8). GSK3β-inhibitor treatment rescued the decrease in mature spine density in *Tbr1* CKO mutants (Figures S8C and S8F). Furthermore, IF analysis of excitatory and inhibitory synaptic densities from the *Tbr1^layer5^* CKO mPFC (layer 5; Figures S8A and S8B) and from the *Tbr1^layer6^* CKO SSCx (layer 6; Figures S8D and S8E) showed a nearly complete rescue of synaptic density 24 h after treatment (Figure S8).

Thus, GSK3β inhibitor treatment of *Tbr1^layer5^* and *Tbr1^layer6^* CKO mice at P60 rescues dendritic spine density as well as excitatory and inhibitory synaptic deficit in *Tbr1* mutant neurons of cortical layers 5 and 6, respectively (Figure S8). This provides an additional line of evidence that augmenting WNT signaling is a key mechanism in restoring mature dendritic spine and synaptic density in *Tbr1* mutants.

### LiCl and GSK3β Inhibitor Treatment at P60 Improves Corticothalamic Axonal Arborization in *Tbr1^layar6^* Mutant

Layer 6 corticothalamic neurons extend their axons to the thalamus where they form synapses. Corticothalamic axons enter the thalamus in *Tbr1^layerr6^* CKOs; however, the corticothalamic axonal arborization is reduced in the anteromedial thalamus of *Tbr1^layer6^* CKOs (white arrowheads in Figure 5M) (Fazel Darbandi et al., 2018). Treatment with either LiCl or GSK3β inhibitor rescued this defect after 24 h (yellow arrowheads in Figures 5N and 5O) and 4 weeks (yellow arrowheads in Figure 5P). Quantification of tdTomato pixel intensity in the anteromedial thalamus (boxed region in Figures 5I and 5M) showed a significant increase after treatment (Figure 5, iv). We estimate that LiCl increased the corticothalamic axonal arborization by ~250 μm in 24 h. Axon growth rates in multiple regions of the nervous system and species have been documented to range from 20 to 75 μm/h (equivalent to ~2,000 μm/24 h) (Goldberg, 2003; Lallemend et al., 2012). We postulate that the rescue of the axonal arbors is through enhanced levels of WNT signaling as result of the LiCl or GSK3β inhibitor treatment.

### Evidence that WNT Signaling Promotes Synaptogenesis in *Tbr1* CKOs through an Autocrine Mechanism

Previously, we demonstrated that restoring *in vivo* *Wnt7b* expression in layer 6 pyramidal neurons of *Tbr1^layer6^* CKOs promoted synaptogenesis onto layer 6 neurons (Fazel Darbandi et al., 2018). Here, we have verified this finding and included additional controls (Figures S9A and S9B).

Toward elucidating whether WNT7B functions through autocrine and/or paracrine mechanisms, we used cortical transplantation of *Wnt7b*-expressing cortical interneurons to study synaptogenesis in *Tbr1^layer6^* CKO and control (WT) mice. We introduced medial ganglionic eminence (MGE)-derived cortical interneurons (MGE donor cells from *Nkx2.1-cre*∷*tdTomato*^*f*/+^ background) harboring either a *Wnt7b* expression construct (*pLenti-DlxI12b-Wnt7b-GFP*) or a control vector (*pLenti-DlxI12b-GFP*) into deep cortical layers of *Tbr1^wild-type^* and *Tbr1^layer6^* CKOs at P1; we analyzed excitatory synaptic density in cortex at P30. We quantified excitatory synapses on apical dendrites of WT and *Tbr1^layer6^* CKOs layer 6 neurons, adjacent to the MGE-transplanted cells (tdTomato^+^-GFP^+^) within layer 5 (Figures S9C-S9F). We did not observe a rescue of synapse numbers (Figure S9E). Furthermore, we did not observe an increase of excitatory synapses onto the soma of the transplanted *Wnt7b*-expressing interneurons (Figure S9F). Thus, this experiment provides evidence that WNT7B promotes synaptogenesis in cortical excitatory neurons through a cell-autonomous autocrine mechanism.

### *Tbr1^layer5^* CKOs Exhibit Social Interaction Defects that Are Rescued by LiCl Treatment

We studied motor function, anxiety, and social interaction of *Tbr1^layer5^* mutant mice between P56 and P80. Motor defects were not detected based on speed in an open field or performance on a rotarod (data not shown). To assay social behavior, we measured the time the experimental mouse spent exploring a novel juvenile WT mouse of the same sex. Subsequently, we measured the amount of time the subject mouse spent exploring a novel object. *Tbr1^layer5^* homozygous CKOs exhibited social interaction deficit with a juvenile mouse; we did not observe a social deficit between *Tbr1^layer5^* WT and *Tbr1^layer5^* heterozygous CKOs (data not shown). Loss of *Tbr1* in layer 5 neurons did not affect the amount of time *Tbr1^layer5^* CKOs spent exploring a novel object compared to the WT.

**Figure 6.**
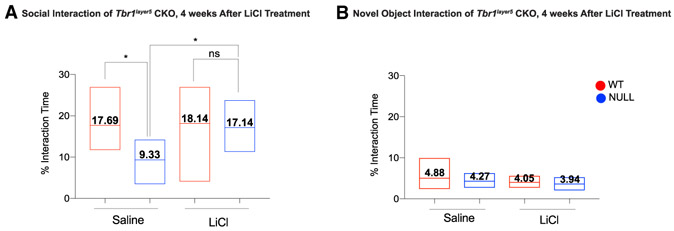
LiCl Treatment Rescues Social Interaction Deficit of *Tbr1^layer5^* Mutants (A) *Tbr1^layer5^* homozygous CKOs (blue) showed reduced social interaction with a juvenile mouse at P56–P80. LiCl treatment of *Tbr1^layer5^* CKOs rescued the social deficit phenotype compared to the saline-treated mutants at P56–P80. (B) LiCl treatment of *Tbr1^layer5^* CKOs did not affect the time spent engaged in novel object exploration compared to the saline-injected control. Floating bar graphs represent the min-max distribution of interaction measured from all genotypes and treatments. Horizontal line in each box denotes the average distribution. Average distribution is numerically indicated in each box. Two-tailed t test with Tukey correction was used for pairwise comparisons (*p < 0.05).

The improved synaptic density of *Tbr1^layer5^* CKOs due to LiCl treatment prompted us to assess the impact of LiCl treatment on the social interaction of *Tbr1^layer5^* CKOs. We performed the novel object exploration and social interaction assays at P60 using *Tbr1^layer5^* WT and CKOs that were treated with a single i.p. injection of saline (control) and LiCl (experimental) 4 weeks prior to the behavioral assays. LiCl treatment of *Tbr1^layer5^* homozygous CKOs improved their social interaction deficit with a juvenile mouse (Figure 6A), while LiCl treatment did not affect a novel object assay (Figure 6B). Thus, LiCl rescues defects in dendritic spines, synapse density, and the social behavior of *Tbr1^layer5^* CKOs.

## DISCUSSION

### *Tbr1* Dosage in Layers 5 and 6 Is Essential for Promoting and Maintaining Dendritic Spine and Synaptic Density

*Tbr1* is expressed in post-mitotic excitatory neurons in the neocortex, hippocampus, entorhinal cortex, pallial amygdala, piriform cortex, olfactory bulb, Cajal-Retzius cells, and subplate neurons (Hevner et al., 2001, 2003). *Tbr1* is best known for its expression and function in layer 6, where it is required to initiate and then maintain layer 6 identity by repressing markers of layer 5 identity (Fazel Darbandi et al., 2018; McKenna et al., 2011). There is also prominent *Tbr1* expression in layer 5 of the rostral cortex, where it is expressed in ~85% of pyramidal neurons (Bulfone et al., 1995).

Here, by deleting *Tbr1* late in gestation using a layer-5-specific Cre (*Rbp4-Cre*), we have investigated the role of *Tbr1* in mPFC development. scRNA-seq from FACS-purified layer 5 neurons of *Tbr1wild-type* and *Tbr1^layer5^* heterozygous and homozygous CKOs demonstrated that *Tbr1* deletion in mPFC layer 5 alters the expression of a subset of genes that control synaptogenesis, synaptic maturation, and microtubule assembly (Tables S1 and S2).

The core phenotypes of the *Tbr1* CKOs are: (1) reduction in the density of mature dendritic spine density (Figures 4 and S6); (2) increased density of immature filamentous (thin) spines (Figure S6); and (3) reduced density of excitatory and inhibitory synapses (Figures 3 and S4). The dendritic spine defect is apparent at the beginning of synaptogenesis (P5) and is maintained through adolescence and into adulthood (Figures 4 and S6). Notably, the *Tbr1* CKOs neurons have an increased I_h_. There is evidence that HCN channels, the mediator of Ih, localize to thin spines (Paspalas et al., 2013). Thus, we hypothesize that the increased I_h_ in *Tbr1* CKOs may be attributed to the increased filamentous spine density in *Tbr1* CKOs. Support for this notion comes from the observation that layer 5 neurons have an ~2-fold increased density of filamentous spines compared to that of layer 6 neurons (Figures S6E and S6F), which correlates with higher I_h_ in layer 5 neurons (Shepherd, 2013).

We postulate that the reduced mature spine density is central to the reduction of excitatory synapses and synaptic activity observed in adolescent (P21) and adult (P56) *Tbr1^layer5^* CKOs. In addition, *Tbr1^layer6^* CKOs (Fazel Darbandi et al., 2018), as well as *Tbr1*^+/−^ constitutive mutants, show defects in dendritic spines and synapses. The fact that we observed defects in dendritic spine and synapse density in *Tbr1* heterozygous CKOs and *Tbr1*^+/−^ constitutive mutants implies that this phenotype could contribute to the behavioral phenotypes in neuropsychiatric disorders such as ASD. This hypothesis is further strengthened by the dendritic spine and synaptic phenotypes in the mPFC, a cortical region with critical functions in cognitive and affective processing.

### Molecular Mechanisms Downstream of *Tbr1* that Promote Synapse Development

**Figure 7.**
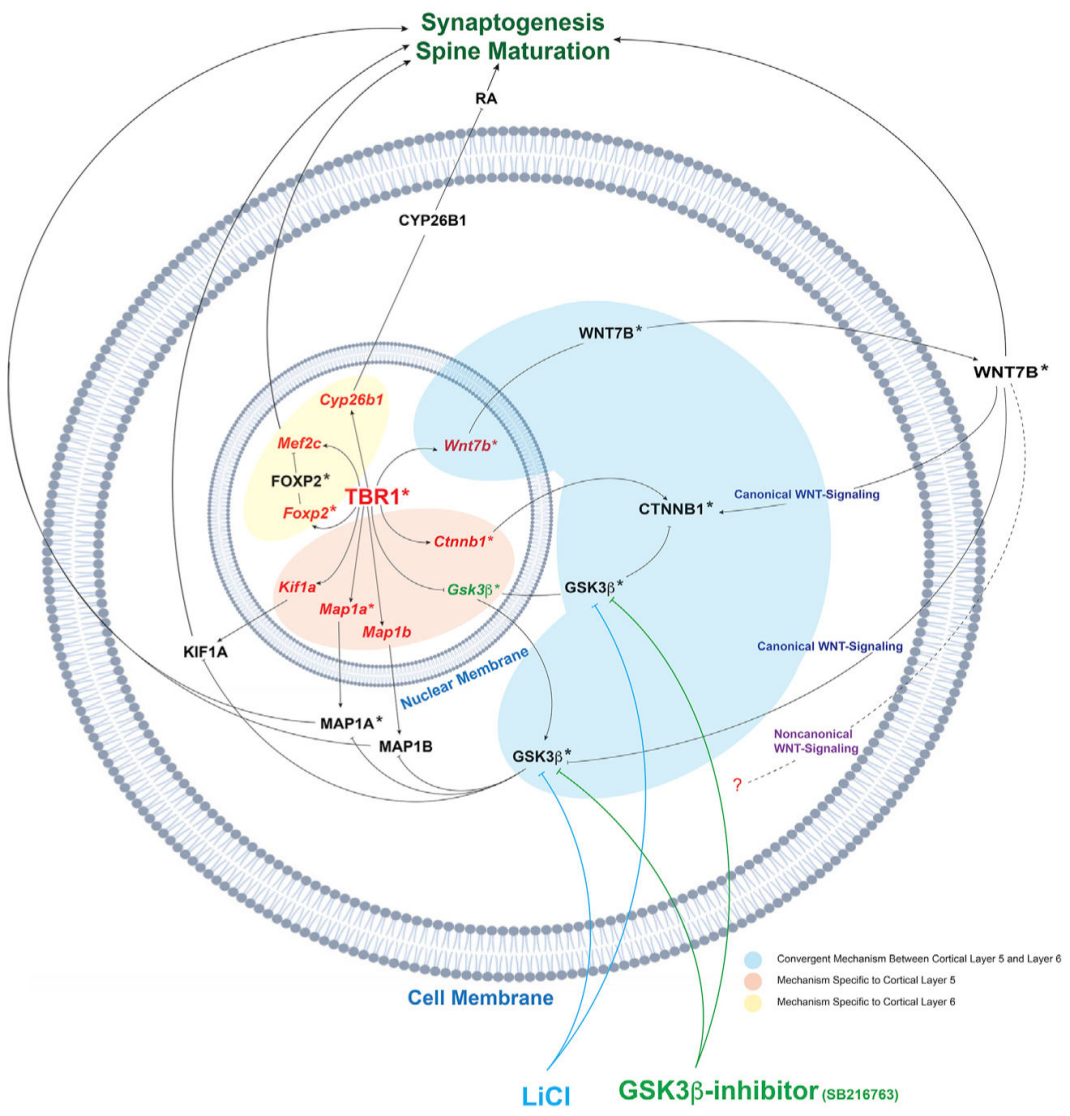
Model of How *Tbr1* Controls Spine Maturation and Synaptogenesis through Promoting WNT Signaling: Links to ASD Pathogenesis Schematic representation of how *Tbr1* controls spine maturation and synaptogenesis in cortical layers 5 and 6. *Tbr1* regulates WNT signaling by promoting *Wnt7b* and *Ctnnb1* and represses *Gsk3β* expression. LiCl (blue) and GSK3β inhibitor (green) rescues *Tbr1* phenotypes through stimulating WNT signaling by inhibiting GSK3β activity. WNT inhibition of GSK3β results in phosphorylation of MAP1A and MAP1B, which promotes microtubule assembly and axonal outgrowth. *Tbr1* activates *Kif1a*, a kinesin motor protein involved in synaptic vesicle trafficking. Furthermore, *Tbr1* activates *Foxp2* and *Mef2c* in layer 6 pyramidal neurons. *Mef2c* promotes the development of excitatory synapses. Lastly, TBR1 promotes expression of *Cyp26b1* in layer 6 pyramidal neurons, which controls RA levels and impacts synaptic development. Asterisks indicate hcASD (red, reduced in *Tbr1* mutants) and pASD (green, increased in *Tbr1* mutants) genes that are involved in these pathways. Cell membrane and nuclear membrane (blue) are indicated. Pathways unique to layer 5 and layer 6 are shown in orange and yellow, respectively. Convergent pathways between layers 5 and 6 are highlighted in blue.

We have evidence that TBR1 controls synaptic development by promoting spine maturation and synaptogenesis through several mechanisms. *Tbr1* promotes WNT signaling (discussed more extensively later), and TBR1 directly drives the expression of *Cyp26b1*, *Foxp2*, *Mef2c*, and *Wnt7b* in layer 6 (Fazel Darbandi et al., 2018), as well as *Kif1a* and *Wnt7b* in layer 5. We integrated these findings into a molecular model (Figure 7). The model also postulates how LiCl and GSK3β inhibitor treatments, through promoting WNT signaling, rescues synaptic and axonal phenotypes in *Tbr1* mutants (Figure 7).

*Tbr1* promotes the expression of *Foxp2* (a hcASD gene) and *Mef2c* transcription factors (TFs) in layer 6 (Fazel Darbandi et al., 2018). *Mef2c* promotes the development of excitatory synapses (Harrington et al., 2016). However, restoring *Mef2c* expression in *Tbr1* mutant neurons failed to rescue their synaptic deficit, suggesting that decreased expression of this TF alone does not underlie the synaptic deficits in *Tbr1* mutants.

*Tbr1* also promotes the expression of *Cyp26b1*, a gene encoding a retinoic-acid (RA)-degrading enzyme, in layer 6 pyramidal neurons. Restoring *Cyp26b1* expression in primary cortical cultures from *Tbr1^layer6^* CKOs rescued synaptic deficit *in vitro* (Figures S9E and S9F). RA acts via RARα in synapses to promote protein synthesis (Chen et al., 2014; Chen and Napoli, 2008). This suggests that *Tbr1*’s control of RA levels, via *Cyp26b1*, can impact synaptic development (Figure 7).

While these three mechanisms appear to contribute to *Tbr1*’s orchestration of synapse development, we believe that *Tbr1*’s control of WNT signaling may be the overriding *Tbr1*-dependent mechanism (Figure 7).

### *Tbr1* Promotion of WNT Signaling Drives Dendritic Spine Maturation and Synaptogenesis on Layer 5 and Layer 6 Pyramidal Neurons

WNT signaling is essential in postsynaptic differentiation of excitatory synapses by recruiting NMDA receptors via promoting PSD95 clustering and local activation of CaMKII within dendritic spines (Ciani et al., 2011). Furthermore, CaMKII is required for WNT-mediated spine growth and increased synaptic strength, thus promoting postsynaptic maturation and differentiation (Ciani et al., 2011). Moreover, WNT expression increases microtubule unbundling and stability by signaling through the canonical pathways downstream of GSK3β (Ciani et al., 2004). WNT inhibition of GSK3β results in phosphorylation of microtubule-associated proteins such as MAP1B. This interaction is essential for microtubule assembly, axonal arborization and outgrowth (Ciani et al., 2004).

Transcriptomic and ISH analyses demonstrate that *Tbr1* promotes expression of *Wnt7b* and *Ctnnb1* (β-catenin) and represses expression of *Gsk3β*. *Wnt7b* encodes a WNT ligand of the canonical WNT signaling pathway (Rosso et al., 2005). *Ctnnb1* encodes β-catenin, the central intracellular signaling protein of the canonical WNT signaling pathway (Ciani and Salinas, 2005). GSK3β is a ubiquitously expressed kinase that represses the canonical WNT pathway by targeting β-catenin for ubiquitin-mediated proteasomal degradation (van Noort et al., 2002). Restoring *Wnt7b* expression rescued the synaptic deficit in *Tbr1^layer6^* mutant neurons *in vitro* and *in vivo* (Fazel Darbandi et al., 2018). To test whether *Wnt7b* is acting through an autocrine or paracrine mechanism, we introduced cortical interneurons ectopically expressing *Wnt7b* into the deep layers of *Tbr1^layer6^* CKOs. We measured their effect on synapse density onto apical dendrites of WT and *Tbr1^layer6^* CKO layer 6 neurons (Figure S9). Because we did not find a statistically significant increase in synapse density, we surmise that WNT7B primarily promotes synaptogenesis cell autonomously onto layer 6 pyramidal neurons.

Furthermore, restoring *Kif1a* expression in layer 5 pyramidal neurons rescued synapses in the *Tbr1^layer5^* CKOs in primary cultures of the neonatal cortex. *Kif1a* is a member of the kinesin family and functions as an anterograde motor protein that controls vesicle delivery in the assembly and function of synapses (Guedes-Dias et al., 2019). GSK3β phosphorylation of kinesins inhibits their activity and thereby reduces anterograde dendritic transport (Gottschalk et al., 2017; Morfini et al., 2002). *De novo* *KIF1A* mutations in human have been associated with intellectual disability (Ohba et al., 2015; Yoshikawa et al., 2018) and hereditary spastic paraplegia (Pennings et al., 2020). In *Drosophila*, loss-of-function mutations in *KIF1A* homolog *Unc-104* causes defects in synaptic transmission by disrupting the formation of mature boutons (Zhang et al., 2017). Thus, the rescue of the dendritic spine and synaptic deficits in *Tbr1* mutants via LiCl and GSK3β-in-hibitor treatments could be, in part, attributed to the enhanced activity of KIF1A proteins as a result of reduced GSK3β activity.

### LiCl and GSK3β Inhibitor Rescue Defects in Dendritic Spine and Synaptic Density in *Tbr1* Mutants

To further explore the hypothesis that reduced WNT signaling in *Tbr1* mutants underlies the reduction in synapses, we tested whether a canonical WNT-signaling pathway agonist, LiCl or GSK3β inhibitor, could rescue dendritic spine and synapse defects. Among LiCl’s best validated mechanisms of action is inhibition of GSK3β, a central kinase in the WNT/β-catenin and AKT pathways (Lenox and Wang, 2003).

LiCl or GSK3β-inhibitor treatment (within 24 h) rescued the dendritic spine density of *Tbr1* mutant neurons in cortical layers 5 and 6. Furthermore, either LiCl or GSK3β-inhibitor treatment rescued excitatory and inhibitory synapse numbers within 24 h. Remarkably, a single dose of LiCl at P30 led to a sustained rescue of synaptic density, measured 4 weeks after treatment. These results suggest that the *Tbr1* mutant’s dendrites have most of the machinery needed to make synapses but have a deficit of the essential signal(s) to initiate synaptogenesis. Once the LiCl- or GSk3β-inhibitor-induced synapses are formed, they appear to be relatively stable.

Corticothalamic axons in the *Tbr1^layer6^* mutants fail to fully arborize within the anterior and anteromedial regions of the thalamus (Fazel Darbandi et al., 2018). This phenotype was also rescued within 24 h of LiCl or GSK3β-inhibitor treatment, suggesting that the reduced WNT signaling underlies the defect of axonal elongation and/or arborization in *Tbr1^layer6^* mutants.

In sum, we postulate that *Tbr1* mutant layer 5 and layer 6 cortical neurons have reduced WNT signaling that underlies their defects in dendritic spines, synapses, and axonal arborization. LiCl or GSK3β inhibitor rescues each of these defects, perhaps through promoting WNT signaling.

### LiCl Treatment Rescues Social Interaction Deficit in *Tbr1^layer5^* CKOs

We eliminated *Tbr1*’s function in cortical layer 5 pyramidal neurons. In most cortical areas, a minority of layer 5 neurons express TBR1, whereas in rostral areas, including the PFC, TBR1 is expressed in ~85% of layer 5 excitatory neurons (Figure S1). The PFC has a central function in distributed circuits that control higher cognitive and emotional functions that are disrupted in neuropsychiatric disorders such as ASD. *Tbr1^layer5^* CKOs are viable and fertile, allowing us to study the impact of *Tbr1* deletion on the behavior of heterozygous and homozygous CKOs. The *Tbr1^layer5^* CKOs showed no deficit in their motor functions (rotarod and open field) and interest in novel objects. However, *Tbr1^layer5^* homozygous CKOs showed a reduction in social interaction with a juvenile mouse. This phenotype had previously been demonstrated in mice with *Tbr1* haploinsufficiency (Huang et al., 2014).

Importantly, treating *Tbr1^layer5^* CKOs with LiCl at P30 rescued the social deficit of *Tbr1^layer5^* CKOs (measured at P56–P80). Thus, perhaps the LiCl-mediated rescue of synaptogenesis may underlie the rescue of the social behavior phenotype. In studies of multiple neuropsychiatric phenotypes, face-valid rodent behavior has, so far, not proven to be a reliable assay for therapeutics development in humans (Sestan and State, 2018). However, the observation here is notable in that it links a risk-specific mutation to an identifiable molecular mechanism and circuit level behavior, offering important traction for future investigations of ASD pathophysiology.

### Insights into How *Tbr1* May Contribute to ASD Pathogenesis

Co-expression network analysis suggests that the *de novo* mutations of ASD-risk genes are enriched in excitatory projection neurons of cortical layers 5 and 6 in the PFCs during human mid-fetal development (Willsey et al., 2013), cell types that also express *Tbr1*. The functions of many ASD-risk genes converge on pathways that control synaptogenesis, synaptic development, and plasticity (Sanders et al., 2015). Thus, in this study, we deleted *Tbr1* in the excitatory neurons of mouse layer 5 of the mPFC at a stage similar to human mid-fetal development.

Our single-cell transcriptomic analysis of FACS-purified layer 5 neurons from the mPFC revealed that *Tbr1* regulates other ASD genes, including *Ank2*, *Ap2s1*, *Ctnnb1*, *Dpysl2*, *Map1a*, *Rorb*, *Smarcc2* (orthologs of high-confidence ASD [hcASD] genes), and *Gsk3β* (ortholog of a probable ASD [pASD] gene) in either *Tbr1^layer5^* heterozygous or homozygous CKOs. *Tbr1^layer5^* heterozygous and homozygous CKOs demonstrated a decrease in dendritic spines and excitatory and inhibitory synaptic densities and reduced sEPSCs and sIPSCs, phenotypes that are convergent with *Tbr1^layerr6^* CKOs, and constitutive *Tbr1*^+/−^. This suggests that decreased TBR1 dosage in human may also impair synaptic development and thereby increase the risk for ASD. While some of the other phenotypes detected in *Tbr1^layer5^* mutants were only present in the homozygotes, including defects in social interaction, these observations could have relevance for ASD, as they denote biological processes that could be altered in *Tbr1* heterozygotes.

### *Tbr1* and *Shank3* Mutants Convergently Present Synaptic and Physiological Defects

The complex genetic variation underlying ASD has complicated efforts to understand the mechanism associated with ASD pathology and therapies. A possible solution for such complex diversity is to identify core mechanisms, in which ASD-risk proteins may act convergently on a common pathway (State and Sestan, 2012). Many mutations are thought to predispose to idiopathic ASDs by causing primary impairments in synaptic transmission (Rosti et al., 2014; Sanders et al., 2015).

Reduced or increased *Shank* expression in *Drosophila* reduces WNT signaling and excitatory synapses (Harris et al., 2016). In mouse, reduced *Shank3* impairs synaptic function by reduction in dendritic arborization, excitatory synaptic density, synaptic transmission, and I_h_ current (Yi et al., 2016). Similarly, *Tbr1* CKOs have evidence for reduced WNT signaling and have reduced mature spine density and excitatory synaptic density (Fazel Darbandi et al., 2018). Likewise, *Tbr1* CKOs have abnormal I_h_ currents in cortical layer 5 (Figure S5) and layer 6 (Fazel Darbandi et al., 2018), although, in *Tbr1* CKOs, I_h_ is increased. TBR1 binds to the *Shank1*, *−2*, and *−3* loci (P2 TBR1 ChIP-seq data; GEO: GSE119362) (Fazel Darbandi et al., 2018), although there are only subtle changes in *Shank* RNA expression in the *Tbr1* mutants. Thus, synaptic dysfunction and, perhaps, reduced WNT signaling are common features of mouse *Tbr1* and *Shank3* mutants; these defects may be the core pathophysiology of some forms of ASD.

### LiCl as a Therapy for Neurodevelopmental Disorders that Have Reduced Synapse Development

Currently, there are no treatments for ASD that address its core biological defects. The ability to restore synapse numbers following lithium administration in the *Tbr1* mutant mice provides an insight to a possible human therapy, especially given that LiCl has a long history of clinical use.

Our study suggests the value of future study of LiCl as a potential treatment for ASD patients with *TBR1* mutations. If successful, LiCl could also conceivably prove relevant for ASD syndromes beyond individuals with TBR1 mutations, particularly where reduced synaptic development is a central feature. In a clinical case report, LiCl was reported to reverse clinical regression, stabilize behavioral abnormalities, and restore brain functioning in two *SHANK3* patients with ASD (Serret et al., 2015). Additionally, it is plausible that the mechanisms identified here could be relevant for patients with *Ank2*, *Ap2s1*, *Ctnnb1*, *Dpysl2*, *Map1a*, *Rorb*, *Smarcc2*, and *Gsk3β*. We also showed that *Tbr1^layer6^* CKOs had arborization defects of their corticothalamic axons that were improved with LiCl, suggesting that LiCl could also improve presynaptic defects. This is consistent with the evidence that WNT signaling positively regulated presynaptic and postsynaptic development (Ahmad-Annuar et al., 2006; Stamatakou and Salinas, 2014).

It is critically important that any hypothesis regarding novel treatments in ASD be subjected to rigorous blinded clinical testing. This is particularly the case for an agent such as LiCl, which has well-known long-term side effects and a narrow therapeutic window. Open-label trials of novel compounds to treat core symptoms in ASD have repeatedly shown promising results early (Choi et al., 2011), only to be followed almost uniformly by negative well-controlled trials. The foregoing consideration of potentially relevant biological mechanisms should not be construed as an immediate clinical recommendation but rather as a justification for additional in-depth and rigorous studies.

Finally, it is remarkable that LiCl in *Tbr1* mutant mice restores dendritic spine density, synaptogenesis, and axon arborization. LiCl has a rapid action (24 h); furthermore, the effect of a single dose lasts over 4 weeks. However, there were many features of the *Tbr1* mutants that did not appear to be rescued by LiCl, including increased layer 5 and layer 6 filamentous spine density and layer 6 dendritic morphogenesis. Thus, while LiCl may have some promise as a therapy, it is improbable that it would fully rescue normal brain function of ASD patients with *TBR1* mutations.

## STAR★METHODS

### LEAD CONTACT AND MATERIALS AVAILABILITY

All unique/stable reagents generated in this study are available from the Lead Contact, Dr. John L. Rubenstein (john.rubenstein@ucsf.edu), without restrictions.

### EXPERIMENTAL MODEL AND SUBJECT DETAILS

#### Animals

All procedures and animal care were approved and performed in accordance with the University of California San Francisco Laboratory Animal Research Center (LARC) guidelines. All strains were maintained on a C57BL/6 background. Animals were housed in a vivarium with a 12hr light, 12hr dark cycle. Postnatally, experimental animals were kept with their littermates. For timed pregnancies, noon on the day of the vaginal plug was counted as embryonic day 0.5.

The *Tbr1^flox^* allele was generated by inGenious Targeting Laboratory (Ronkonkoma, NY). LoxP sites were inserted into introns 1 and 3, flanking *Tbr1* exons 2 and 3 (Fazel Darbandi et al., 2018). To enable selection of homologous recombinants, the LoxP site in intron 3 was embedded in a *neo* cassette that was flanked by *Flp* sites. The *neo* cassette was removed by mating to a *Flp*-expressing mouse to generate the *Tbr1^flox^* allele. Cre excision removes exons 2 and 3, including the T-box DNA binding region, similar to the constitutive null allele (Bulfone et al., 1998). *Rbp4-cre* mice (Gensat KL100) were used to delete *Tbr1* in layer 5 projection neurons. *tdTomato*^*fl*/+^ (*Ai14*) mice were crossed with *Tbr1^f/f^* mice and used as an endogenous reporter. *Tbr1* layer 5 knockout mice (*Tbr1^layer5^* mutant) were generated by crossing *Tbr1^f/f^*∷*tdTomato*^*f*/+^ mice with *Tbr1*^*f*/+^∷*Rpb4-cre*^+^. The specific gender and age of experimental animals can be found in the Results section and corresponding figure legends.

### TRANSGENIC ANIMAL MODELS

The mouse strains used for this research project, B6.FVB(Cg)-Tg(Ntsr1-cre)GN220Gsat/Mmucd, RRID:MMRRC_030648-UCD and B6.FVB(Cg)-Tg(*Rbp4-cre*)KL100Gsat/Mmucd, RRID:MMRRC_037128-UCD, were obtained from the Mutant Mouse Resource and Research Center (MMRRC) at University of California at Davis, an NIH-funded strain repository, and was donated to the MMRRC by MMRRC at UCD, University of California, Davis. Made from the original strain (MMRRC:032081) donated by Nathaniel Heintz, Ph.D., The Rockefeller University, GENSAT http://gensat.org/index.html and Charles Gerfen, Ph.D., National Institutes of Health, National Institute of Mental Health.

Information about the generation and genotyping of the transgenic lines used in this study can be found in the corresponding original studies: *Rbp4-Cre* (Gong et al., 2007), lox-STOP-lox-tdTomato (Ai14;(Madisen et al., 2010)). Mice were maintained on C57BL/6J background.

### METHOD DETAILS

#### Genomic DNA extraction and genotyping

Tissue samples were digested in a solution containing 1 mg/mL of proteinase K, 50 mM Tris-HCl pH 8.0, 100 mM EDTA, 100 mM NaCl and 1% SDS. Genomic DNA was extracted using a standard ethanol precipitation protocol. Genotyping was performed with PCR-based assays using purified genomic DNA, and primer-pair combinations flanking the deleted region and detecting *Cre* and *tdTomato* alleles.

#### RNA extraction and cDNA synthesis

Total RNA was extracted from the cortices of wild-type mice at P0 using RNeasy Plus® Mini Kit (QIAGEN) following the manufacturer’s protocol. First strand cDNA was synthesized using Superscript reverse transcriptase II following manufacturer’s protocol (Thermofisher). cDNA library was used as template to clone and generate *in situ* probes.

#### Single-Cell RNA-sequencing (scRNA-seq) on FAC-Sorted Cells

Layer specific transcriptome profiling was conducted by using 10X Chromium scRNA-seq on FAC-Sorted cells from medial prefrontal cortex of *Tbr1^wild-type^* and *Tbr1^layer5^* heterozygous and homozygous mutants at P5. The medial prefrontal cortex was dissected in HBSS from P5 mice. Cortices were dissociated using a Papain Dissociation System (Worthington Biochemical Corporation) following manufacturer’s protocol. tdTomato^+^ cells were sorted using BD FACS Aria II Cell Sorter at Center for Advanced Technology (UCSF). Approximately 20,000 *tdTomato*^+^ cells were collected from each sample. Following FAC-sorting, the cell suspensions were centrifuged at 300 × g for 5 min. Cells were washed for a total of 3 times with 1 mL 1X PBS supplemented with 0.04% BSA. Following the final wash, the cell pellet was resuspended with 25 μL of 1X PBS supplemented with 0.04% BSA. Cell concentration for each sample was determined using trypan blue and a hemocytometer. We targeted to capture approximately 5000 cells per each genotype to generate scRNA-seq libraries. Single cell RNA-seq was performed using 10X Chromium Single Cell 3′ Reagent Kit v2 following manufacturer’s protocol. Library concentration was assessed with Qubit dsDNA HS Assay Kit following manufacturer’s protocol (Thermofisher). Library fragment size distribution was examined on the Agilent Bioanalyzer 2100 (Agilent Technologies) and Agilent High Sensitivity DNA Kit (Agilent Technologies) following manufacturer’s protocol. Libraries were sequenced on Hiseq4000 at Center for Advanced Technology (UCSF).

#### Computational Analysis of FAC-Sorted Layer 5 scRNA-Seq data

##### Read pre-processing

Single cell RNA-sequencing libraries were sequenced on Illumina Hiseq4000 to an average depth of 45K reads per cell. Read quality control, UMI counting, barcode counting, and alignment to the mouse reference genome (mm10) were performed using the “cell-ranger 2.0.1” pipeline provided by the manufacturer.

##### Filtering and Normalization

The initial dataset contained 17,823 cells with an average of 892 genes per cell. Cells with greater than 30% of mitochondrial genes were removed as this is indicative of poor-quality cells (n = 82). Cells with fewer than 500 or more than 10,000 unique-molecular-identifier (UMI) counts were removed as this often represents sequencing errors (n = 163). Cells with fewer than 500 or more than 3,000 genes were removed based on the distribution (n = 182). Genes which occurred in less than 0.01% of cells were also removed (n = 13065). The remaining 17,396 cells and 14,933 genes were used for downstream analysis. No experimental factors were determined to explain a disproportionate of expression variance using the Single Cell Analysis Toolkit for gene Expression in R (scater; v 1.9.15).

Using the R package Seurat (v 2.3.4), the data was log normalized for each cell by the total expression and scaled to 10,000 transcripts per cell. Variable genes were identified using the *FindVariabieGenes()* function which calculates the average expression and dispersion for each gene, then bins genes and calculates a z-score for dispersion within each bin. The data was scaled, centered, and regressed on the percent of mitochondrial gene content, number of UMI counts, and the number of genes.

##### Cell-type Identification and Clustering

TSNE was generated using all principal components accounting for more than 2% of the variance and a clustering resolution of 0.3 which resulted in 12 clusters (average silhouette width 0.16). Three clusters were identified as neuronal cells using known markers *Nrgn*, *Rorb*, and *Cnih2*.

The raw data from the three identified neuronal clusters was retained and filtered again based on the distribution of UMI counts and the number of genes per cell (N = 11,943). We applied more stringent filtering to genes by removing mitochondrial genes, ribosomal genes, pseudogenes, genes that did not occur in 1% of neuronal cells, and genes with a variation below the median variation across all genes (N = 7,174). The data normalized as described above and TSNE was generated using all principal components accounting for more than 4% of the variance and a clustering resolution of 0.3 which resulted in 6 clusters (average silhouette width 0.15). Two clusters were identified as atypical cells due to a reduced expression in excitatory neuronal markers and subsequently removed from downstream DEX analysis.

##### Differential Gene Expression (DEX) Analysis and Gene Ontology Enrichment

To identify gene signatures of each genotype, we used MAST and the zero-inflated regression (zlm) method to compare raw UMI counts (i.e., non-normalized counts) per gene across the cells in the population (FDR < 0.05). Genes that pass a 0.05 significant threshold are considered as significantly differentially expressed (DEX) genes. Gene Ontology enrichment analysis of common differentially expressed genes was performed using the R package goseq (v 1.34.1) using all expressed genes (N = 7,174) as background.

##### Data and Code Availability

The data used in this publication have been deposited in NCBI’s Gene Expression Omnibus (GEO) under accession number GSE146298 (https://www.ncbi.nlm.nih.gov/geo/query/acc.cgi?acc=GSE146298). In addition, all the scripts that were used for analyzing scRNA-seq data as well as the result files are available on https://github.com/aseveritt/Darbandi_TBR1_L5scRNAseq.

#### Primary Cell Culture and *in vitro* Rescue Assay

##### Primary Cell Culture

Cortex was dissected from P0 *Tbr1^wild-type^* and *Tbr1^layer5^* homozygous mutants and dissociated using papain dissociation kit following manufacturer’s protocol (Worthington). A total of 300,000 cells were seeded into tissue culture slides pre-coated with poly-L-lysine (10 mg/ml, Sigma) and then laminin (5 mg/ml, Sigma), and grown *in vitro* with media containing DMEM-H21 with 5% fetal bovine serum for 3 hr. After the cells recovered, DMEM-H21 media was replaced by Neurobasal medium containing B27 supplement, 25% glucose, and glutamax overnight.

##### In vitro *Rescue Assay*

*Syt4*, *Mef2c*, *Kif1a* and *Rac3* cDNA was cloned into pcDNA3.1(−) (Thermofisher Scientific). *Tbr1^layer5^* mutant cells were transfected with *Syt4*, *Mef2c*, *Kif1a*, *Rac3* expression vectors and *Tbr1^wild-type^* were transfected with mock empty vector using Lipofectamine 3000 (Invitrogen) for 6 hr. Following incubation, the media was replaced by Neurobasal medium containing B27 supplement, Penicillin/Streptomycin, 25% glucose, and glutamax. Cultures were grown for 14 days *in vitro*. After 14 days, cultures were washed 3 times with 0.5 mL 1X PBS for 5 min each and fixed for 15 min with 4% PFA in 1X PBS at RT. Fixed cells were washed 3 times with 0.5 mL 1X PBS and blocked in 1X PBS containing 10% Normal Serum, 0.1% Triton X- 100 and 2% BSA for 1 hr at RT. Primary antibodies including mouse anti-Vglut1 (1:200, Synaptic Systems) and rabbit anti-PSD95 (1:200, Cell Signaling; excitatory synapses), rabbit anti-Vgat (1:500, Synaptic Systems) and mouse anti-gephyrin (1:200, Synaptic Systems; inhibitory synapses) were diluted 1:200 in blocking solution. Cells were stained for excitatory and inhibitory synapses with primary antibodies for 48 hr at 4°C with gentle shaking. On a shaker, the cells were washed 3 times with 0.5 mL 1X PBS for 5 min each and incubated with the secondary antibody for 2 hr (room temperature), washed 3X with 1X PBS, and mounted. This experiment was repeated twice (n = 2).

#### *In vivo* Synapse Rescue Assays

We performed *in vivo* rescue assay of synaptic deficit in *Tbr1* mutant mice using three different approaches. First, we directly injected a lentivirus harboring WNT7B. Second, we utilized a transplantation assay to deliver the protein of interest (WNT7B) by introducing MGE progenitor cells, following previously published MGE transplantation assay (Vogt et al., 2015). Lastly, we used a single intraperitoneal injection of LiCl to rescue the decrease in synapse numbers in *Tbr1* mutants.

##### Direct lentiviral injection

*In vivo* rescue assay was carried out by cloning *Wnt7b* into a *Cre*-dependent lentiviral backbone (*pLenti*-*CAG-Flex-IRES-GFP*). *CAG-Flex-GFP* (empty vector) and *Wnt7b-IRES-GFP* expressing lentivirus (*pLenti-CAG-Flex-Wnt7b-IRES-GFP*) were generated in HEK293T cells as previously reported (Vogt et al., 2015) using Polyplus jetPRIMEH® transfection reagent following manufacturer’s protocol.

Lentivirus containing *CAG-Flex-GFP* or *Wnt7b-IRES-GFP* were injected in the SSCx of *Tbr1^layer6^* wild-type as well as *Tbr1^layer6^* heterozygous and homozygous CKO pups at P1. For injections, a glass micropipette of 50 μm diameter (with a beveled tip) was preloaded with sterile mineral oil and viral suspension was front-loaded into the tip of the needle using a plunger connected to a hydraulic drive (Narishige) that was mounted to a stereotaxic frame. P1 pups from *Tbr1^layer6^* wild-type and *Tbr1^layer6^* heterozygous and homozygous CKOs were anesthetized on ice for 1–2 min before injections. Each pup received 2–3 viral injections (150 nL per site) in the right hemisphere. These sites were about 1 mm apart along the rostral to caudal axis. Viral suspensions were injected into layer 6 of the neonatal SSCx. After injections, pups were put back with the mother to recover after they began to move around on their own. Mice were sacrificed 21 days after injection and transcardially perfused with PBS followed by 4% PFA.

##### MGE-Derived Interneuron Transplantation Assay

A detailed protocol for the MGE transplantation assay has been previously described (Vogt et al., 2015). First, E13.5 MGEs from *Nkx2.1-cre*∷*tdTomato*^*f*/+^ embryos were dissected in ice-cold HBSS. Next, cells were mechanically dissociated by repeated pipetting (10–15 times) through a 1000 μL plastic pipette tip in DMEM media that contained 10% fetal bovine serum. Cells were dissociated in DMEM with 10% FBS that was preconditioned in a tissue culture incubator at 37°C and with 5% CO_2_ to achieve a physiological pH. The cells were then transfected with either *DlxI12b-GFP* (control) or *DlxI12b-Wnt7b-GFP* (WNT7B-GFP expressing). Cells were transfected for 30 min at 37°C then pelleted by centrifugation (3 min, 700 × g), and resuspended in 2-3 μL of DMEM, put on ice, and then remaining media containing the transfected MGE cells was removed before loaded into the injection needle. For injections, a glass micropipette of 50 μm diameter (with a beveled tip) was preloaded with sterile mineral oil and cells were front-loaded into the tip of the needle using a plunger connected to a hydraulic drive (Narishige) that was mounted to a stereotaxic frame. *Tbr1^layer6^* Wild-type and *Tbr1^layer6^* homozygous CKO P1 pups were anesthetized on ice for 1-2 min before being placed into a molded surface (modeling clay) for injections. Each pup received 2-3 injections of cells (~100 nL per site) in the right hemisphere. These sites were about 1mm apart along the rostral to caudal axis; cells were injected into layers 5/6 of the neocortex. After injections, pups were put back with the mother to recover after they began to move around on their own. Mice were sacrificed 28 days after transplantation and transcardially perfused with PBS followed by 4% PFA.

##### Lithium chloride (LiCl) injection

P59 and P30 mice were administered a single intraperitoneal (IP) injection of 400 mg/kg LiCl or saline in a volume of 4 ml/kg (Martin et al., 2018). Treated mice were anesthetized at P60, 24 hr or 4 weeks after LiCl injection with intraperitoneal injection of 100 mg/kg Ketamine containing 15 mg/kg Xylazine. A separate cohort of P58 mice were administered a single IP injection of 400 mg/kg LiCl or saline in a volume of 4 ml/kg. Treated mice were anesthetized 24 hr after LiCl injection with intraperitoneal injection 100 mg/kg Ketamine containing 15 mg/kg Xylazine. All brains were processed at P60. Animals were perfused transcardially with ice-cold 1X PBS and then with 4% PFA in 1X PBS, followed by brain isolation, 1-2 hr post-fixation, cryoprotected in 30% sucrose in PBS, and cut frozen (coronally or sagittally) on a sliding microtome at 40μm for immunohistochemistry.

#### Histology

For P0 and P3 experiments, neonatal animals were anesthetized on ice. For P21 and P56 experiments, animals were anesthetized with intraperitoneal injection of 100 mg/kg Ketamine containing 15 mg/kg Xylazine. Animals were perfused transcardially with cold PBS and then with 4% PFA in PBS, followed by brain isolation, 1-2 hr post-fixation, cryoprotected in 30% sucrose in PBS, and cut frozen (coronally or sagittally) on a sliding microtome at 40μm for immunohistochemistry or *in situ* hybridization. All primary and secondary antibodies were diluted in PBS containing 10% Normal Serum, 0.25% Triton X-100 and 2% BSA. The following primary antibodies were used: mouse anti-Vglut1 (1:200, Synaptic Systems), rabbit anti-Vgat (1:500, Synaptic Systems), rabbit anti-PSD95 (1:200, Cell Signaling), mouse anti-gephyrin (1:200, Synaptic Systems). The secondary antibodies for immunofluorescence were Alexa Fluor-conjugated and purchased from Thermofisher. For *in vivo* synapse immunohistochemistry, a total of n = 30 apical dendrites were counted from each of *Tbr1^wild-type^*, *Tbr1^layer5^* heterozygous and *Tbr1^layer5^* homozygous mutants. The coronal sections were pre-treated with pepsin to enhance the staining. Immunofluorescence specimens were counterstained with 1% DAPI to assist the delineation of cortical layers. For *in situ* hybridization a rostro-caudal coronal series of at least ten sections from n = 2 brains from *Tbr1^wild-type^* and *Tbr1^layer5^* heterozygous and homozygous mutants were examined. Anti-sense riboprobes for *Calm2*, *Kif1a*, *Wnt7b*, and *Mgst3* were prepared as previously described (Cobos et al., 2005; Fazel Darbandi et al., 2016). We also investigated cortical lamination within rostral cortex including PFCx of wild-type brain at P3 and P21 using anti-sense riboprobes for lamination markers *Cux2*, *Rorb*, *Etv1*, *Tbr1* and *Nr4a2*. ISH was performed using digoxigenin-labeled riboprobes.

#### Image Acquisition and Analysis

Fluorescent and bright-field images were taken using a Coolsnap camera (Photometrics) mounted on a Nikon Eclipse 80i microscope using NIS Elements acquisition software (Nikon). Confocal imaging experiments were conducted at the Cancer Research Laboratory (CRL) Molecular Imaging Center, supported by Helen Wills Neuroscience Institute at UC Berkeley. Confocal images were acquired using Zeiss LSM 880 with Airyscan with a 63X objective at 1,024 × 1,024 pixels resolution with 2.0X optical zoom using ZEN 2.0 software. Brightness and contrast were adjusted, and images merged using Photoshop or ImageJ software. ImageJ software was used for image processing. For synapse counting (presynaptic and postsynaptic boutons), confocal image stacks (0.4μm step size) were processed with ImageJ software. In brief, background subtraction and smooth filter were applied to each stack. Using a threshold function, each stack was converted into a ‘masks’ image. Furthermore, the channels were co-localized with the Image Calculator plugging. Lastly, the number of co-localizations were counted, and the length of each dendrite was measured in each of the focal plane. Staining for control and mutant were done in parallel as well as the image capturing.

#### Electrophysiology

Coronal brain slices (250 μm) including medial prefrontal cortex were made from three mice (n = 3) at age p21-28 and at p56-p80. Slicing solution was chilled to 4°C and contained (in mM): 234 sucrose, 26 NaHCO_3_, 11 glucose, 10 MgSO_4_, 2.5 KCl, 1.25 NaH_2_PO_4_, 0.5 CaCl_2_, bubbled with 5% CO_2_/ 95% O_2_. Slices were incubated in artificial cerebrospinal fluid (aCSF) at 32°C for 30 minutes and then at room temperature until recording. aCSF contained (in mM): 123 NaCl, 26 NaHCO_3_, 11 glucose, 3 KCl, 2 CaCl_2_, 1.25 NaH_2_PO_4_, 1 MgCl_2_, also bubbled with 5% CO_2_/ 95% O_2_. Neurons were visualized using differential interference contrast or DODT contrast microscopy on an upright microscope (Olympus). *Rbp4-cre* positive neurons were identified by fluorescent visualization of cre-dependent tdTomato. We obtained somatic whole-cell patch clamp recordings using a Multiclamp 700B (Molecular Devices) amplifier and acquired with pClamp. Patch pipettes (2-5 MΩ tip resistance) were filled with the following (in mM): 130 KGluconate, 10 KCl, 10 HEPES, 10 EGTA, 2 MgCl_2_, 2 MgATP, 0.3 Na_3_GTP. All recordings were made at 32-34°C. Series resistance was compensated in all current clamp experiments and monitored throughout recordings. Recordings were discarded if Rs changed by > 25%. For spontaneous EPSC and IPSC recordings cells were held in voltage clamp at −70 mV and +10mV, respectively. In both cases patch pipettes were filled with the following (in mM): 135 Cesium Methanesulfonate, 8 NaCl, 10 HEPES, 0.3 EGTA, 5 QX314, 4 MgATP, 0.3 Na_3_GTP.

#### Behavioral Assays

Experiments were conducted during the light cycle (8am to 8pm). Mice were habituated to investigator handling for 1-2min on three consecutive days. On the testing day, mice were transferred to experimental room and allowed to habituate for at least 45 minutes prior to testing. All behavior assays were performed on mice age P56 to P80. We were blind to the genotypes during scoring of videos.

##### Open-field test

An individual mouse was placed near the wall-side of 50 × 50 cm open-field arena, and the movement of the mouse was recorded by a video camera for 10 min. The recorded video file was analyzed with Any-Maze software (San Diego Instruments). Time in the center of the field (a 25 × 25 cm square) was measured. The open field arena was cleaned with 70% ethanol and wiped with paper towels between each trial.

##### Elevated plus maze test

An individual mouse was placed at the junction of the open and closed arms, facing the arm opposite to the experimenter, of an apparatus with two open arms without walls (30 × 5 × 0.5 cm) across from each other and perpendicular to two closed arms with walls (30 × 5 × 15 cm) with a center platform (5 × 5 cm), and at a height of 40 cm above the floor. The movement of the mouse was recorded by a video camera for 10 min. The recorded video file was analyzed with Any-Maze software and time in the open arms of the apparatus was measured. The arms of the elevated plus maze apparatus was cleaned with 70% ethanol and wiped with paper towels between each trial.

##### Rotarod test

The assay consisted of four trials per day over the course of 2 days with the rotarod set to accelerate from 4rpm to 45rpm over 5 minutes. The trial started once five mice were placed on the rotarod rotating at 4rpm in separate partitioned compartments. Each trial ended when a mouse fell off, made three complete revolutions while hanging on, or reached 300 s. Digital videos of the mice on the rotarod were recorded from behind. The rotarod apparatus was cleaned with 70% ethanol and wiped with paper towels between each trial.

##### Social interaction and novel object task

An individual mouse was allowed to habituate for 5 minutes in their home cage prior to starting the trial. A juvenile (3-4 weeks old) mouse of the same strain and sex was introduced to the home cage. After 5 minutes, the juvenile was removed from the home cage. After a 5 min break a novel object (typically a plastic test tube cap) was introduced into the home cage for five minutes. We scored videos offline, blind to genotype. We measured the number of seconds the mouse spent with its nose in direct contact with the novel object or engaged in social interaction with the juvenile (defined as sniffing, close following, or allo-grooming) in the 300 s following the time the juvenile or object was introduced into the cage. In addition, we noted any aggressive-appearing behaviors toward the juvenile, freezing, and grooming behaviors. We repeated this behavioral assay on adult wild-type and mutant mice that were treated with a single IP injection of LiCl and compared to vehicle treated animals injected with saline.

### QUANTIFICATION AND STATISTICAL ANALYSIS

All individual data points are shown as well as mean ± SEM. All statistical analyses were performed using GraphPad Prism 7.0 software. Statistical significance was accepted at the level p < 0.05. We used Student’s t test to compare pairs of groups if data were normally distributed (verified using Lillie test). If more than two groups were compared, we used one-way ANOVA with post hoc tests between groups corrected for multiple comparisons (Holm-Sidak or Tukey). Forthe ISH experiments reported in this paper n = 2 represents two biological replicates for each of the reported genes. We examined the changes in synapse numbers of n = 30 different dendrites from n = 2 animals for each genotype. Whole-cell patch clamp experiments at P21 and P56 were conducted from n = 3 different animals for each age and genotype. Lastly, behavioral analysis was conducted from n = 11/8/9, wild-type/ heterozygous/homozygous animals. The specific n for each experiment as well as the post hoc test, exact F and corrected p values can be found in the Results section.

### DATA AND CODE AVAILABILITY

Data and MATLAB analysis scripts are available upon request from the Lead Contact.

## Supplementary Material

Supp Info 1

Supp Info 2

Supp Info 3

Supp Info 4

5

## Figures and Tables

**Table:** KEY RESOURCES TABLE

REAGENT or RESOURCE	SOURCE	IDENTIFIER
Antibodies		
Rabbit anti-Vglutl polyclonal antibody	Synaptic Systems	Cat# 135303
		RRID: AB_887875
Mouse anti-PSD95 antibody	NeuroMab (UC Davis)	Cat# 75-028
		RRID: AB_2307331
Rabbit anti-Vgat polyclonal antibody	Synaptic Systems	Cat# 131002
		RRID: AB_887871
Mouse anti-Gephyrin polyclonal antibody	Synaptic Systems	Cat# 147011
		RRID: AB_887717
Goat anti-Rabbit IgG Alexa Fluor 488	Thermofisher Scientific	Cat# A-11008
		RRID: AB_143165
Goat anti-Mouse Alexa Fluor 647	Thermofisher Scientific	Cat# A32728
		RRID: AB_2633277
Bacterial and Virus Strains		
pLenti- CAG- Flex-Wnt7b-IRES-GFP	This paper	N/A
pLenti-DlxI12b-Wnt7b-GFP	This Paper	N/A
pLenti-DlxI12b-GFP	This Paper	N/A
pLenti-CAG-Flex-IRES-GFP	This Paper	N/A
Chemicals, Peptides, and Recombinant Proteins		
Sucrose	Sigma Aldrich	Cat# S5016
Sodium bicarbonate (NaHCO_3_)	Sigma Aldrich	Cat# S6014
Glucose	Sigma Aldrich	Cat# G5767
Magnesium sulfate (MgSO_4_)	Sigma Aldrich	Cat# 230391
Sodium phosphate monobasic monohydrate (NaH_2_PO_4_)	Sigma Aldrich	Cat# P9638
Potassium chloride (KCl)	Sigma Aldrich	Cat# P9333
Calcium chloride dehydrate (CaCl_2_)	Sigma Aldrich	Cat# 223506
Magnesium chloride dexahydrate (MgCl_2_)	Sigma Aldrich	Cat# M9272
Potassium gluconate (KGluconate)	Sigma Aldrich	Cat# P1847
HEPES	Sigma Aldrich	Cat# H3375
EGTA	Sigma Aldrich	Cat# E4378
Adenosine 5′-triphosphate magnesium salt (Mg-ATP)	Sigma Aldrich	Cat# A9187
Guanosine 5′-triphosphate sodium salt hydrate (Na_3_GTP)	Sigma Aldrich	Cat# 51120
Cesium Methanesulfonate	Sigma Aldrich	Cat# C1426
Sodium Chloride (NaCl)	Sigma Aldrich	Cat# S9888
QX314 chloride	Tocris	Cat# 2313
ZD7288	Tocris	Cat# 1000
Critical Commercial Assays		
Bioanalyzer High Sensitivity DNA Kit	Agilent	Cat# 5067-4626
Bioanalyzer RNA 6000 Nano Kit	Agilent	Cat# 5067-1511
Chromium i7 Multiplex Kit,	10X Genomics	Cat# 120262
Chromium Single Cell 3’ Chip Kit v2	10X Genomics	Cat# 120236
Chromium Single Cell 3′ Library & Gel Bead Kit v2	10X Genomics	Cat# 120237
Deposited Data		
*Tbr1* P5 scRNA-seq Raw and Analyzed Data	This paper	https://www.ncbi.nlm.nih.gov/geo/query/acc.cgi?acc=GSE146298
Experimental Models: Cell Lines		
Mouse primary cortical culture	This paper	N/A
HEK293 cells	Thermofisher Scientific	Cat# R79007
Experimental Models: Organisms/Strains		
Mouse TBR1 conditional mutant	This paper	N/A
Mouse TBR1 constitutive mutant	This paper	N/A
Oligonucleotides		
Primer for genotyping flox allele	This Paper	N/A
ND.for GAC ACA CAC CCT TCT TCA GTT TAC AGC		
Primer for genotyping flox allele	This Paper	N/A
ND.rev CAA GCC CGA CTG CCA ATG TTC TG		
Primer for genotyping Ntsr1-cre allele	This Paper	N/A
Ntsr1-cre.for GAC GCC ACG CCC CCC TTA		
Primer for genotyping Ntsr1-cre allele	This Paper	N/A
Ntsr1-cre.rev CGG CAA ACG GAC AGA AGC ATT		
Primer for genotyping Rbp4-cre allele	This Paper	N/A
Rbp4-cre.for GGG CGG CCT CGG TCC TC		
Primer for genotyping Rbp4-cre allele	This Paper	N/A
Rbp4-cre.rev CCC CAG AAA TGC CAG ATT ACG TAT		
Primer for genotyping tdTomato allele	This Paper	N/A
tdTomato.for CTG TTC CTG TAC GGC ATG G		
Primer for genotyping tdTomato allele	This Paper	N/A
tdTomato.rev GGC ATT AAA GCA GCG TAT CC		
Primer for genotyping constitutive allele	This Paper	N/A
LN.for CAT TCA GAG CGA CGC ATC AAA GC		
Primer for genotyping constitutive allele	This Paper	N/A
LN.rev CAA GCC CGA CTG CCA ATG TTC TG		
Recombinant DNA		
For complete list of recombinant DNA, please refer to Table S5.	This Paper	N/A
pcDNA3.1(−)	Thermofisher	Cat# V79520
Software and Algorithms		
ImageJ	N/A	https://imagej.nih.gov/ij/
MiniAnalysis	http://www.synaptosoft.com/MiniAnalysis/	v6.0.7
GraphPad Prism	https://www.graphpad.com/scientific-software/prism/	v7.01
MATLAB	https://www.mathworks.com/products/matlab.html	v8.6.0.267246
Clampex and Multiclamp	https://www.moleculardevices.com/products/axon-patch-clamp-system/acquisition-and-analysis-software/pclamp-software-suite	v10.2
ANY-maze	https://www.stoeltingco.com/any-maze-video-tracking-software-1224.html	v5
Imaris	https://imaris.oxinst.com/downloads	v9.2.1
Single-cell RNA-seq code availability	This Paper	https://github.com/aseveritt/Darbandi_TBR1_L5scRNAseq

## Highlights

- *Tbr1* promotes and maintains spine maturation and synaptogenesis through WNT signaling
- Promoting WNT signaling rescues dendritic spine and synaptic defects in *Tbr1* mutants
- TBR1 directly regulates transcriptional circuits that control ASD-risk genes
