# Supplementary material for: Enhancing WNT Signaling Restores Cortical Neuronal Spine Maturation and Synaptogenesis in Tbr1 Mutants
Source: Cell Rep. Author manuscript; Available in PMC 2020 Sep 4. (PMC7473600; doi:10.1016/j.celrep.2020.03.059)
Supplement: 5 [file NIHMS1622004-supplement-5.pdf]

Cell Reports, Volume *31*

Supplemental Information

Enhancing WNT Signaling Restores Cortical Neuronal Spine Maturation and Synaptogenesis in *Tbr1* Mutants

Siavash Fazel Darbandi, Sarah E. Robinson Schwartz, Emily Ling-Lin Pai, Amanda Everitt, Marc L. Turner, Benjamin N.R. Cheyette, A. Jeremy Willsey, Matthew W. State, Vikaas S. Sohal, and John L.R. Rubenstein

Table S5: Related to Figure 2. Complete list of DNA FASTA sequences that were cloned to generate DIG-labeled RNA probes for ISH experiments.

Recombinant DNA
>*Tbr1*<br>CACTCGCTCTTTCACTTGACCCTCGATGACCGTCTGCGGGGATAAGTGCAGGTCTCTCACTATGATTTTAAAACTCTTCTTTTTT<br>CTTTCTTTCTTTCTCTCTACACAGCCTTCTCTGCAGTTAGCGCACCGACCTTGAACCTGGCTGTAAACCTTGTGGTTTTCCAACTT<br>TTCGTCTGTGAGGTTATGATCCTCCCTGTCTTTTTTCCACCCCCTTCTCCTTGCCCCACTCATCCTCTCCTTTCTCTTGGAATGAA<br>ACTCTTCAACTTTAGGAGACCTGGGCAATCCTGCCAGGCAGCAGCGATTCCGACCCGCCTTGTCTTGGCCTCCCTATTTAACCA<br>TAGGATGTTGACTCTAGAACCTGCACCCACCCAGCGCGTCCTTTCTTATACCCGAGTGGATGGATGGATGGATGGATGGATGG<br>TAGGGATGTTAATACTTTTAGTGGAACAAAGCCTGTGAAATGATTGTATATAGTGTTAATTTATTGTAACGAATGGCTAGTTTT<br>TATTCTCATTGTCAAGGCACAAAACCAGTTCACGCTTAACTTTTTATTCCTTTCCTTTCTTCTCCTTTTCTTTTTCTCCTCTCATTC<br>TTTCTCTTCTCCCACACCCTTTGTTTTCTTGTGAGTTATTAAAGATATTCTAAGAGGCTCTGGAAACACGAAGCACTTCATAGTG<br>GTGGCTTTCTCACTTCTCAGTTCGTTGCATGATGTAACCACTGTGTGCCCTGGTGCACACAATGTAGCTAAGGAGAATCCACCT<br>GAACACCTGTAAAAGCTAGTTGTCTGTTCCTAGGCGAGTCGAGTAAGTGACACGATGCCTGCCAGGCGGACTTAACTGGAGTT<br>CTATGTGTTTCTCCCTTCCTTCTAAATGGAATGGCCCCACATCAGCAATATTATTTTGCCTTATTTGTTTTTCCCCAAAGTGCCA<br>AATCCATTACTGGTCTGTGCAGGTGCCAAATATGCTGATAAACTGTTTCTGACTATCTTTTCAGACCCCACTC
>*Nr4a2*<br>GACAGCAGTCCTCCATTAAGGTAGAAGACATTCAGATGCACAACTACCAGCAACACAGCCACCTGCCCCCTCAGTCCGAGGAG<br>ATGATGCCACACAGCGGGTCGGTTTACTACAAGCCCTCTTCGCCCCCGACACCCAGCACCCCGAGCTTCCAGGTGCAGCATAG<br>CCCGATGTGGGACGATCCGGGCTCCCTTCACAACTTCCACCAGAACTACGTGGCCACTACGCATATGATCGAGCAGAGGAAGA<br>CACCTGTCTCCCGCCTGTCACTCTTCTCCTTTAAGCAGTCGCCCCCGGGCACTCCTGTGTCTAGCTGCCAGATGCGCTTCGACGG<br>GCCTCTGCACGTCCCCATGAACCCGGAGCCCGCGGGCAGCCACCACGTAGTGGATGGGCAGACCTTCGCCGTGCCCAACCCCA<br>TTCGCAAGCCGGCATCCATGGGCTTCCCGGGCCTGCAGATCGGCCACGCATCGCAGTTGCTTGACACGCAGGTGCCCTCGCCG<br>CCGTCCCGGGGCTCTCCCTCCAATGAGGGTCTGTGCGCTGTTTGCGGTGACAACGCGGCCTGTCAGCACTACGGTGTTCGCACT<br>TGTGAGGGCTGCAAAGGTTTCTTTAAGCGCACGGTGCAAAAAAACGCGAAATATGTGTGTTTAGCAAATAAAAACTGCCCAGT<br>GGACAAGCGCCGCCGAAATCGTTGTCAGTACTGTCGGTTTCAGAAGTGCCTAGCTGTTGGGATGGTTAAAGAAGTGGTTCGCA<br>CGGACAGTTTAAAAGGCCGGAGAGGTCGTTTACCCTCGAAGCCGAAGAGCCCACAGGATCCCTCTCCCCCCTCACCTCCGGTG<br>AGTCTGATCAGTGCCCTCGTCAGAGCCCACGTCGATTCCAATCCGGCAATGACCAGCCTGGACTATTCCAGGTTCCAGGCAAA<br>CCCTGACTATCAGATG
>*Etv1*<br>GTGCCTCTGTCTCACTTTGATGAGAGCATGACCTACATGCCCGAAGGGGGCTGCTGCAACCCTCACCCCTACAACGAAGGATA<br>CGTGTACTAACATGAGTAACCCGTCAAGCAAGGCACCCCGTCGTTCGCCTCTTTTTTTTTCAAGATGCAGAGAATCACCGAATT<br>CTCTTCGATGTTTGTTTTATTTCTGTTGTTTGTACTTTATTTTTTAAATGATAATACAAAAGGGGGCTTTTCCTGTTGCATTATTC<br>TATGGTCTGCCATGGACTGCGCACTTTATTTGCTGGTGGGCGGGAGTAATCTAGACATTCATTCTTTGTAACAGGAAGATGGCG<br>GATGAGTGGGCAGAAGGAGCTGAGGGATTCCTTTTTGCTTAGGCTTGGAATGGAGTCCTACAGGTTTCTGTATGATGATGCTAT<br>ATCATATTTGTTCGATTTCATAATAACGTAAGATAATTTTCCCTGGGATCTACGGTACAGTTGATTTCACGTTGTGTAAATATCT<br>TCTTGGAGACCATTTGCCTTGGGCATTTCCCCCATCATTACTGAGTCTCTGCAGGTGTACAAAAATCTACTGTAAATGGCAGTT<br>TAATGTTAGAAATTACTGTTTTGCACCTCTTGTAAAAAAAAAAATATTTAGCAATTGCATTTGTTGTTCTTTCTGTTTCATAATGC<br>TTTACAGATGACTTGTAGAGGAAAACCCTAAATGTGGGCAATTCTCTCTGAAGTTGAGTAATCACCATGACTGTAAATGAGGG<br>CCACCGTTTTGGACTCTGGCTCCAACTGAGTCACAGGCCAGTA
>*Rorb*<br>GCACAGAACATCATTAAGTCCCATTTGGAGACATGTCAGTACACCATGGAAGAACTCCATCAGCTGGCATGGCAGACCCACAC<br>CTACGAGGAAATCAAGGCGTATCAAAGCAAGTCCAGGGAGGCTCTGTGGCAGCAGTGTGCCATCCAGATCACCCATGCTATCC<br>AGTACGTGGTGGAGTTCGCCAAGCGGATAACAGGCTTCATGGAGCTGTGTCAGAACGATCAGATCTTACTTCTGAAGTCAGGT<br>TGCTTGGAAGTGGTTTAGTGAGAATGTGTCGTGCCTTCAACCCATTAAACAACACTGTTCTGTTTGAAGGAAAATATGGAGG<br>AATGCAAATGTTCAAAGCCTTAGGTTCGGATGACCTAGTGAATGAAGCATTTGACTTTGCGAAGAATCTGTGTTCCTTGCAGCT<br>GACTGAGGAAGAGATTGCTCTGTTCTCCTCTGCTGTTCTGATATCCCCAGACCGAGCCTGGCTGATCGAACCAAGAAAAGTCC<br>AGAAGCTTCAGGAAAAGATTTATTTTGCACTGCAACATGTGATTCAGAAGAACCACCTGGATGATGAGACCCTGGCAAAGTTA<br>ATAGCCAAGATACCAACTATCACGGCAGTCTGCAACTTGCATGGGGAGAAGCTGCAGGTATTTAAGCAGTCTCATCCAGACAT<br>AGTGAATACACTGTTTCCTCCATTGTACAAGGAGCTCTTTAATCCTGACTGTGCTGCGGTCTGCAAATGAAGGGGACGAGAAC<br>TCTCAGAGTCATGGAATGCATCGCCGTTAAGACAAAAGCAATGTGTTCATGGGGACTTAAGGAAAATGTCACTACTGCAACAT<br>TAGGAATGTCCTGCACTTAATAGAAATATTTTTCACCGCTACAGTTTGAAGAATGTAAATATGCACCTGAGTGGGGCTCTTTTG<br>TTTGTTCGTTTGTTGTTTGTTTGTTTTTGAAATGATCATAAATATACAAATATAGGACACTGGGTGTTATCTTTTTTAATTTTATT<br>CGGGTATGTTTTGGAGACAACTG

>*Cux2*

GAGCTGGACACATACTCCATCACCAAGAGAGTCAAGGAGGTCCTCACCGACAACAACCTAGGGCAGCGGCTGTTTGGTGAGA
GCATCTTGGGGTTGACCCAGGGCTCCGTGTCAGATCTGCTGTCGAGGCCCAAGCCCTGGCACAAACTGAGCTTGAAGGGCCGG
GAGCCCTTTGTGCGTATGCAGCTGTGGCTGAGTGACCCCCACAACGTGGAGAAGCTTCGGGACATGAAGAAGCTGGAGAAGA
AAGCCTATCTGAAGCGCCGCTATGGGCTCATCGGCACCGGCTCGGACAGCGAGTCACCGGCTGCGCACTCCGAGTGCCCCAGC
CCGTGTTTGCAGCCCCAGGAGTTGAGTCTCATGCAGGCCAAGAAGCCCAGGGTGGTGCTGGCGCCCGCCGAGAAGGAGGCTCT
GCGGAAGGCCTACCAGCTCGAGCCGTACCCCTCGCAGCAGACCATAGAGCTGCTCTCCTTCCAACTCAACCTCAAGACGAACA
CCGTCATCAACTGGTTCCACAACTACAGGTCCAGGATGCGCCGTGAAATGCTGGTGGAGGGGACACAGGATGATCCTGACTTT
GACCCGAGTGGGGGTCCCAATGTCCTGACGCCAGGCCACACCCACAGAGAGCCCACCCCACAGAGCCCCGACTCAGAGACTG
AGGACCAAAAGCCCCCCATGAAGAGCTTAGAGCTGCAAGAGCCTGAGGGTCCCCTACAGCGAGCTGCCCCAGACAGGGCTCT
GGTGAAGATCAAACAGGAAGAGGGTTTGGAGGTGGATGGAGACAGCCAGCCCCAGGATGTGGGGGATCCAGACCGAGGGCA
AGATGGCCCCAAAGAGGAGCATACCCACCCTCTGGGAAACAGTGACCTCTCAGAGCTGGCCCCAGGGCCCTTTCTTTCAGGCA
CACCCAATCCAGACTGTCCTTCATTGCACAACCCCCAAGAAAAGGGGACTGGGGAACAGGTTCACTCAGAGCCTCTGAGTTTC
AAGTCCA

>*Mgst3*

GGTGAGCCAGAGCCAAGATGGCTGTCCTCTCTAAGGAGTATGGATTTGTGCTTCTCACTGGTGCTGCCAGCTTTGTGATGGTGC
TCCACCTAGCCATCAACGTGGGCAAAGCCCGCAAGAAGTACAAGGTAGAGTACCCTGTCATGTACAGCACAGATCCTGAGAA
CGGGCATATGTTCAACTGCATTCAGCGCGCCCACCAGAACACGTTGGAGGTGTACCCTCCCTTCCTGTTTTTCCTAACGGTGGG
AGGTGTTTACCACCCGCGCATAGCTTCTGGCCTGGGCCTGGCCTGGATTATTGGGCGAGTCCTTTACGCATATGGCTACTACAC
AGGAGACCCTAGCAAGCGGTATCGAGGAGCCGTGGGCTCTCTTGCCCTCTTTGCCCTGATGGGCACCACCGTGTGCTCTGCTTT
CCAGCATCTCGGCTGGATCAGACCAGGCTTAGGCTACGGGTCCAGATCCTGCCACCACTGAGGTGTGGAGGGCCTTCCGACTC
TCACTCACCTCCAGCGACTCACCCTGA

>*Wnt7b*

ACGCAATGGTGGTCTGGTACCCAATGGAGACAAATCCCTTTACTTCTCTTTGGGAAAGTGAACCACAAAGGGACCATGAGACT
CTGAGGGTCACCTCCCTGCCTGTGACTGGACACAGAAAGGCCACACCCACCAGTCACACTCAAAACGGTTTCCTGGGCTGTTT
CCTGCCGGCCCTGGGCAGTGTGGATGGATGTTGACAAAATTATTTATGTTTTCTTAGCATCAGATGAGGACTCAGTACTAACGA
CTGGGTAGCCAGACCTAACCCTATTTGAGGACACCCTTCCCTCACTCCTCCCGGCCCCTCCCTGCAGGGTCCTCTGCTCCTTGC
AGAACTCGAGGATGTCAGAATTGGCACGGAAGCTGGCTGGTGGGGGGACTCCTTATCAGCACCTTGGGAGGGGCTTGGTGGC
CCTACAAGGCCTGAGATGGCCGCAGAGGACAGCCAATCTTCCATTCCATTTGGAGACTGTCATGCAAATCAAATGTCCCTTGT
GTCAGGCTCCAGGCATGCCTCGTCCTCTCCCTGGTCCTTCACCCTCCCAGCCTGCTGCCAACCTCCACCTCCAGTTTACAAATTC
TCTTCTCCTCTGGAGCCAACCTGACACCCAGGACTGCCCCACAGGTTCAGGAGAGGTCAGGGACAGTTGCCCCACATGACAGA
TGGACAGAGGGCAATCTGAAGATTTACTGGAGACCCCACGGCTCTGTGAAATAAATATACTGACACAGCCCCATCCAGCCCAA
CTCTGGAAGTTGCCAGGGTGATGGGAGGCTGCACCCCCTTTTCAGTACCTTGGGTTTTGTCCTTCTTCTGTGATCCTGATGCCAG
AGAACTGACATCCAGAATTTAGGGATGTATTGGTCAGGCCCCCTGCCTAGTGTCCACTGATACCTGCTTCAGGGTCCTTATATT
ATGAGGACATGGGACCCTCAAACAGGGGTCCGTGGGAAGCTTAATGTCCCATTTCCTCAGGCCCT

>*Calm2*

CTGACCAACTGACTGAAGAGCAGATTGCAGAGTTCAAAGAAGCTTTCTCACTATTTGACAAGGACGGGGATGGGACAATAAC
AACAAAGGAGCTGGGGACAGTGATGAGGTCCCTGGGGCAGAACCCCACAGAAGCAGAACTGCAGGACATGATCAATGAAGT
AGATGCGGATGGTAATGGCACAATTGATTTCCCTGAATTTCTGACAATGATGGCAAGAAAAATGAAAGACACAGACAGTGAA
GAAGAGATTAGAGAAGCATTCCGTGTGTTTGATAAGGATGGCAATGGCTATATTAGTGCAGCAGAGCTTCGCCATGTGATGAC
AAACCTTGGCGAGAAGTTAACAGATGAAGAGGTCGATGAGATGATCAGGGAAGCAGACATTGACGGGGACGGTCAGGTAAA
CTACGAAGAGTTTGTACAAATGATGACAGCGAAGTGAAGACATTGTACAGAATGTGTTAAATTTCTTGTACAAAATTGTTTATT
GCCATTTCTTTGTTTGTAACTTATCTGTAAAAGGTTCCCCCCCCCTACTGTCAAAAAATATGCATGTATAGTAATTAGGACTTCA
TTCCTCCATGTTTTCT

>*Kif1a*

CTTCGGAAATGACACTAGGACCTTCTACCAGTTTGAAGCAGCCTGGGACAGTTCCATGCATAACTCTCTCCTGCTGAATCGTGT
CACCCCTTACCGGGAGAAAATCTACATGACGCTCTCTGCCTACATTGAGATGGAGAACTGTACCCAGCCAGCTGTCATCACCA
AGGACTTCTGCATGGTTTTCTACTCTCGGGACGCCAAGCTACCCGCCTCACGCTCCATTCGCAACCTGTTTGGCAGTGGCAGCC
TGCGGGCCACAGAAGGCAACCGTGTGACTGGTGTGTATGAGCTCAGCCTTTGCCACGTGGCCGACGCAGGCAGCCCAGGGAT
GCAGCGTCGGCGCCGGCGGGTGCTGGACACATCAGTAGCCTATGTCCGGGGTGAGGAGAACCTGGCTGGCTGGAGGCCCCGA
AGTGACAGCCTGATCCTAGATCACCAGTGGGAACTGGAGAAACTGAGCCTCTTACAGGAGGTGGAAAAGACCAGGCACTACC
TGCTCCTTCGGGAGAAGCTGGAGACGACCCAGCGCCCTGGCCCTGAGGTGCTGTCCCCAGCCTCCAGTGAGGACTCTGAGTCC
CGTAGTTCTTCAGGTGCCTCTTCCCCATTGTCAGCCGAGGGTCAGCCGTCACCTCTGGAGGCTCCCAACGAGAGGCAGAGGGA
GCTGGCTGTCAAGTGCTTACGACTCCTCATGCACACATTCAACAGGGAATACACC

Figure S1: Related to Figure 1. TBR1 expression in mPFC of P5 and P21 Layer 5; TBR1 genomic binding; *Mgst3* – a new layer 5 marker.

(A) Immunohistochemistry (IHC) was used to determine the overlap between TBR1 and *Rbp4-cre::tdTomato*$^{f/+}$ reporter in wildtype mPFCx at P5 and P21. Scale bar = 100 μm.

(B) Quantification of tdTomato^{+}/TBR1^{+} cells in wildtype mPFCx at P5 and P21. Neonatally (P5) ~60% and postnatally (P21) ~85% of reporter^{+} cells (layer 5 excitatory neurons) are TBR1^{+}. Error bars represent SEM of TBR1^{+}/tdTomato^{+} cells in wildtype mPFCx at each age.

(C) TBR1 genomic binding (ChIP-Seq) on wildtype whole cortex at P2 (red tracks). Red boxes represent the TBR1 binding that reached statistical significance. TBR1 directly regulates a subset of genes involved in spine maturation and synaptogenesis, including *Ctnnb1*, *Gsk3β*, *Kif1a, Map1a, Map1b,* and *Wnt7b*. TBR1 also directly regulates *Mgst3*, a new layer 5 marker. Genes are shown in blue. Black arrow indicates the direction of transcription. Genomic scale (in Kb) are shown for each locus. **(D)** *In situ* hybridization demonstrates the expression pattern of *Mgst3* in wildtype brain at P3. At this age, *Mgst3* is a layer 5 specific marker in the neocortex. Cortical layers 2-4, 5^{upper}, 5^{lower}, 6 and 6b (subplate) are labeled. Scale bar = 500 μm.

A) TBR1 IHC in Wildtype mPFCx

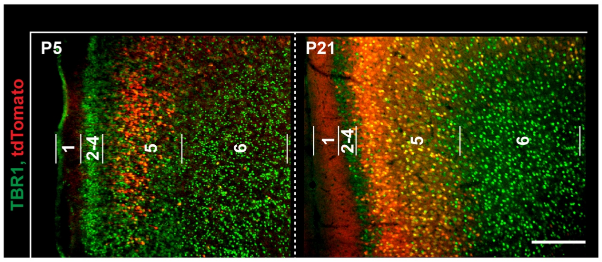

B) Quantification of TBR1⁺/tdTomato⁺ in Wildtype mPFCx

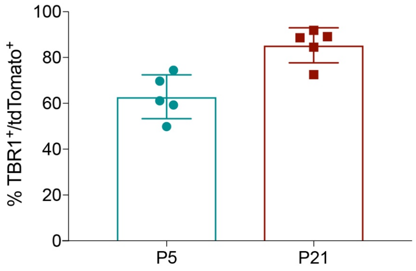

C) TBR1 ChIP-Seq Coverage of *Tbr1*-Regulated Genes at P2

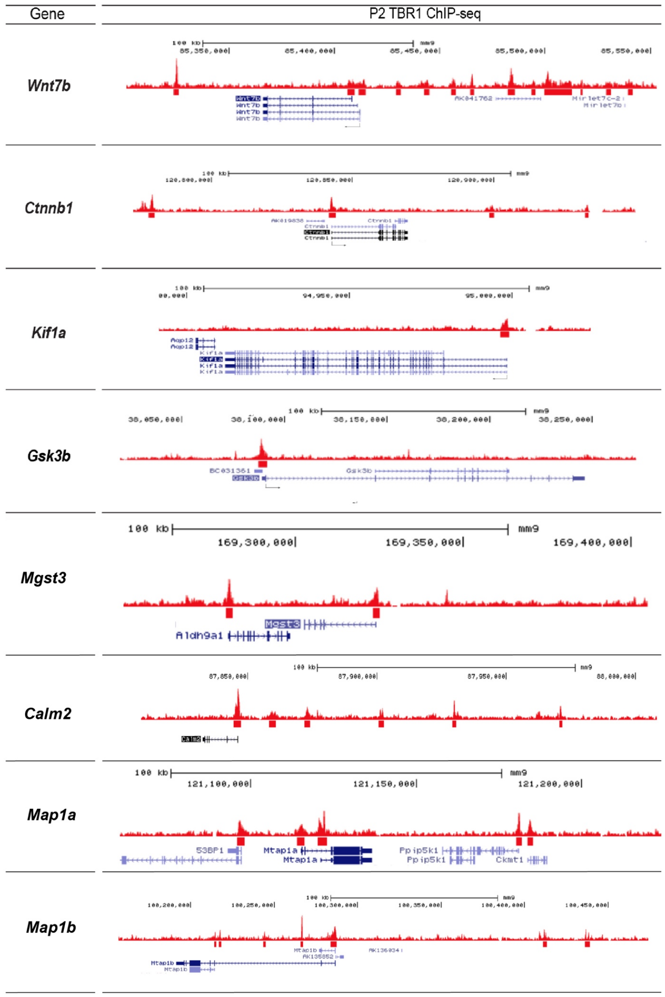

D) Wildtype *Mgst3* Expression

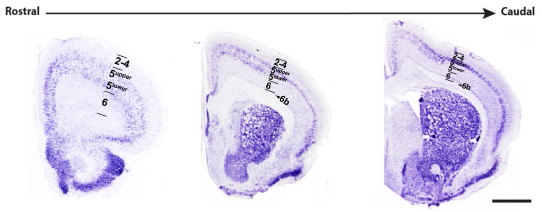

Figure S2: Related to Figure 1. Filtering and clustering of single-cell RNA-seq.
(A) Histograms of the three metrics (number of unique molecular identifiers, number of genes, fraction of mitochondrial genes) used to filter low quality cells from single-cell data. Red line denotes thresholds. **(B)** Boxplots displaying the fraction of mitochondrial content per cell for both publication and initial pilot experiments suggest 30% is an ideal threshold for this dataset. The pilot experiment data was not included due to lower coverage and lack of *Tbr1*^{layer5} heterozygous CKOs. **(C)** Thresholds were adapted to better fit the neuronal cell population. **(D)** tSNE plot displaying 17,396 single cells colored by identified cell type and **(E)** genotype show cell-type is stronger predictor than library preparation. All experiments for the three genotypes was performed simultaneously and run on the same 10x chip. **(F)** tSNE plot displaying 11,070 single cells colored by genotype.

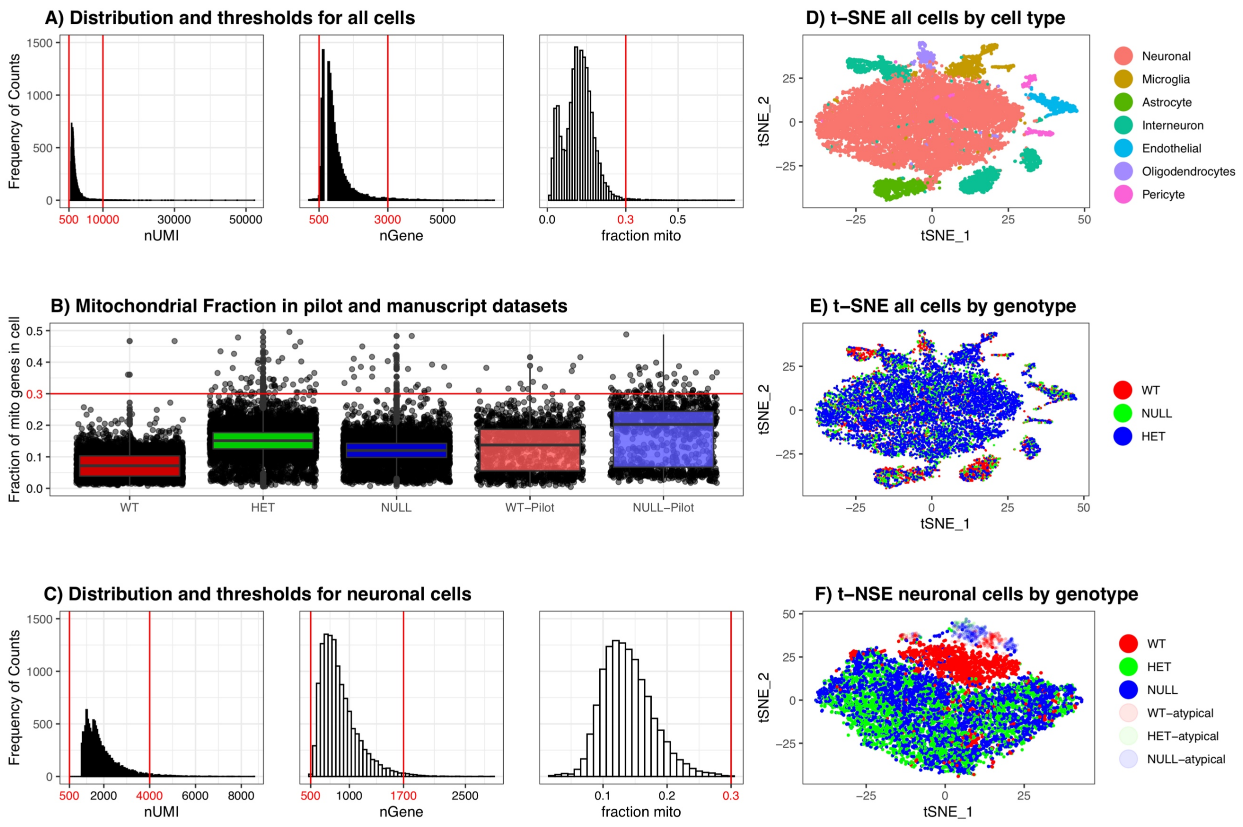

A) Distribution and thresholds for all cells
Frequency of Counts
nUMI
nGene
fraction mito
D) t–SNE all cells by cell type
Neuronal
Microglia
Astrocyte
Interneuron
Endothelial
Oligodendrocytes
Pericyte
tSNE_1
tSNE_2
B) Mitochondrial Fraction in pilot and manuscript datasets
Fraction of mito genes in cell
WT
HET
NULL
WT–Pilot
NULL–Pilot
E) t–SNE all cells by genotype
WT
NULL
HET
C) Distribution and thresholds for neuronal cells
F) t–NSE neuronal cells by genotype
WT
HET
NULL
WT–atypical
HET–atypical
NULL–atypical

Figure S3: Related to Figures 1 and 2. Classical cortical layer 5 markers expressed in single-cell data and expression plots for select differentially expressed genes.

(i) Feature plots displaying the normalized expression of select markers *Nrgn*, *Bcl11b*, *Foxp1*, and *Neurod6* across the neuronal cell population. **(ii)** Density plots showing the distribution of expression values for select DEX genes in neuronal across genotypes. *Calm2* and *Mgst3* are upregulated in *Tbr1*^{layer5} homozygous CKO, while *Kif1a* and *Cox7b* are downregulated.

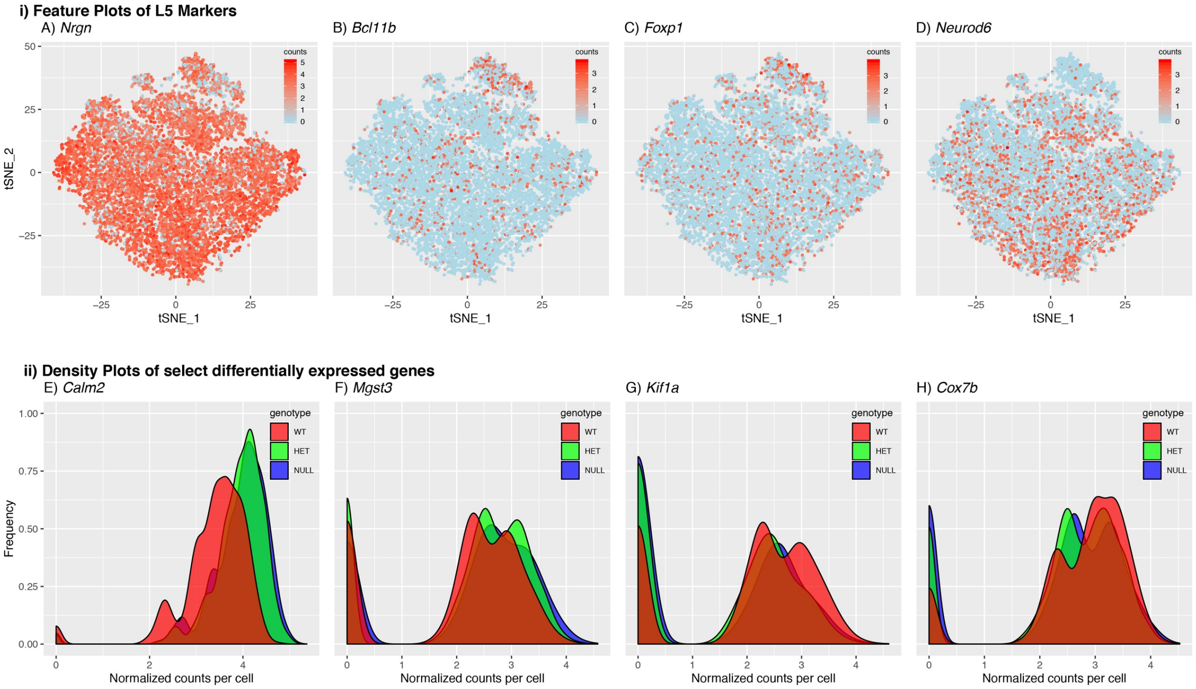

i) Feature Plots of L5 Markers
A) Nrgn
B) Bcl11b
C) Foxp1
D) Neurod6
counts
tSNE_1
tSNE_2
ii) Density Plots of select differentially expressed genes
E) Calm2
F) Mgst3
G) Kif1a
H) Cox7b
genotype
WT
HET
NULL
Frequency
Normalized counts per cell

Figure S4: Related to Figure 3. Loss of *Tbr1* in layer 5 reduces excitatory and inhibitory synaptic input onto the layer 5 pyramidal neurons in mPFCx at P21.

(A) Schematic representation of medial prefrontal cortex (mPFCx, blue boxes). The blue box represents the region of mPFCx utilized for imaging and whole-cell patch clamp experiments. (B, C) Schema of layer 5 projection neuron (red) in mPFCx of $Tbr1^{wildtype}$ (B) and $Tbr1^{layer5}$ CKOs (C). The purple rectangles indicate the zone within layers 2/3 where synapse numbers were analyzed. Pipette tip indicates that the soma was patched during the electrophysiology recordings (B, C).

(i) Excitatory synapses were analyzed via synaptic bouton staining onto apical dendrites of layer 5 neurons (n=30 dendrites) and spontaneous EPSC (sEPSC) recordings from the soma of (D) $Tbr1^{wildtype}$, (E) $Tbr1^{layer5}$ heterozygous, and (F) $Tbr1^{layer5}$ homozygous CKOs at P21 (D-F). *Rbp4-cre::tdTomato*$^{f/+}$ allele was used to label the layer 5 neurons (red). ImageJ software was used to process confocal images for quantification. (G) Quantification of excitatory synaptic density. Excitatory synapse numbers were reduced by 37% in $Tbr1^{layer5}$ heterozygous (BD = 0.680, p<0.0001) and 74% in $Tbr1^{layer5}$ homozygous CKOs at P21 (BD = 0.286, p<0.0001) (H) Sample traces of sEPSC recordings at -70mV in mPFCx slices from $Tbr1^{wildtype}$ (red), $Tbr1^{layer5}$ heterozygous (green), and $Tbr1^{layer5}$ homozygous CKOs (blue) at P21. (I) Quantification of the sEPSC frequency in layer 5 neurons at P21 (n = 6/6/6, wildtype/ heterozygous/ homozygous cells from two different animals/genotype; One-way ANOVA, $F_{(2,15)} = 23.18$, $p < 0.0001$; t-test, Tukey correction, wildtype v. homozygous: $q_{(15)} = 9.416$, $p < 0.0001$; heterozygous v. homozygous: $q_{(15)} = 6.455$, $p = 0.001$).

(ii) Inhibitory synapses were examined by synaptic bouton staining onto apical dendrites of layer 5 neurons (J – L) and spontaneous IPSC (sIPSC) recordings from the soma of the layer 5 neurons of (J) $Tbr1^{wildtype}$, (K) $Tbr1^{layer5}$ heterozygous, and (L) $Tbr1^{layer5}$ homozygous CKOs at P21. *Rbp4-cre::tdTomato*$^{f/+}$ allele was used to label the layer 5 neurons (red). ImageJ software was used to process confocal images for quantification. (M) Quantification of inhibitory synaptic density at P21. Inhibitory synapse numbers were reduced ~26% in $Tbr1^{layer5}$ heterozygous CKOs (BD = 0.816, p<0.0001) and ~71% decrease in $Tbr1^{layer6}$ homozygous mutants (BD = 0.319, p<0.0001). (N) Sample traces of sIPSC recordings in voltage clamp at +10mV in SSCx slices from $Tbr1^{wildtype}$ (red), $Tbr1^{layer5}$ heterozygous (green), and $Tbr1^{layer5}$ homozygous CKOs (blue) at P21. (O) Quantification of the sIPSC frequency in layer 5 neurons at P21 (n = 7/7/7, wildtype/ heterozygous/ homozygous cells from two different animals/genotype; One-way ANOVA, $F_{(2,18)} = 5.159$, $p = 0.0169$; t-test, Tukey correction, wildtype v. homozygous: $q_{(18)} = 4.534$, $p = 0.0129$). Two-way ANOVA was used for the statistical analysis of the control, heterozygous and null. Two-tailed T-test with Tukey correction was used for pairwise comparisons. Floating bar graphs represent the min-max distribution of synaptic density and/or E/IPSC frequency measured from each genotype. Horizontal line in each box denotes the average distribution. Average distribution is numerically indicated in each box. BD = Bouton Density. (*p< 0.05) (**p< 0.01) (***p<0.001) (****p< 0.0001).

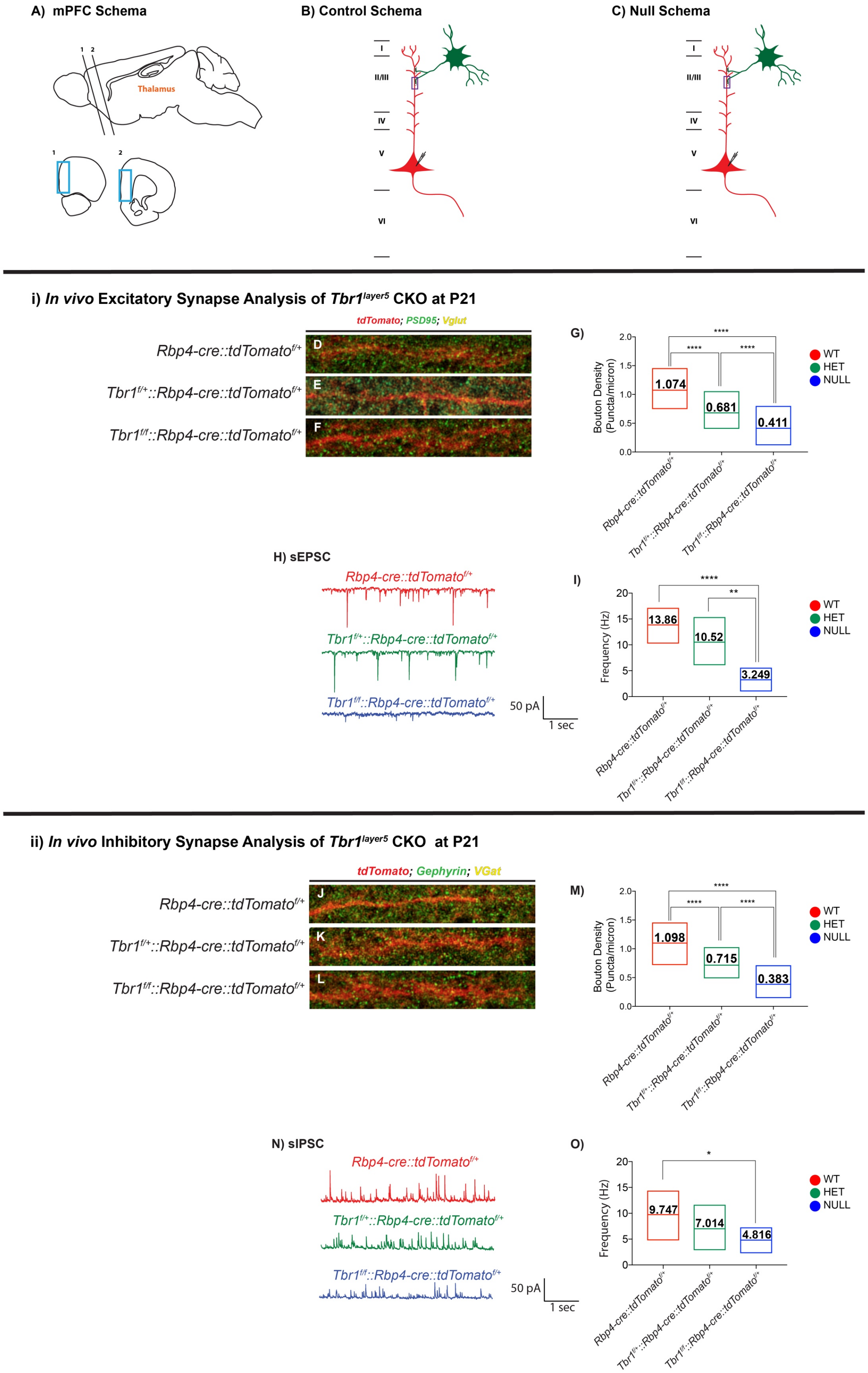

A) mPFC Schema
1
2
Thalamus
B) Control Schema
I
II/III
IV
V
VI
C) Null Schema
i) In vivo Excitatory Synapse Analysis of Tbr1layer5 CKO at P21
tdTomato; PSD95; Vglut
Rbp4-cre::tdTomatof/+
Tbr1f/+::Rbp4-cre::tdTomatof/+
Tbr1f/f::Rbp4-cre::tdTomatof/+
D
E
F
G)
Bouton Density (Puncta/micron)
1.074
0.681
0.411
****
WT
HET
NULL
H) sEPSC
50 pA
1 sec
I)
Frequency (Hz)
13.86
10.52
3.249
**
ii) In vivo Inhibitory Synapse Analysis of Tbr1layer5 CKO at P21
tdTomato; Gephyrin; VGat
J
K
L
M)
1.098
0.715
0.383
N) sIPSC
O)
9.747
7.014
4.816
*

Figure S5: Related to Figure 3. Loss of *Tbr1* in layer 5 prefrontal cortex results in an increase in hyperpolarization-activated cation currents (I_h).

Whole-cell patch clamp recordings from layer 5 mPFCx at P56 **(A-D)** show that many intrinsic electrophysiological properties were unaffected by loss of *Tbr1*, including resting membrane potential **(B)**, input resistance **(C)**, and action potential half-width (data not shown). **(D)** ''Sag and rebound'' is increased in *Tbr1^{layer5}* mutant neurons (n = 7/6/7, wildtype/ heterozygous/ homozygous cells from two different animals/genotype; One-way ANOVA, $F_{(2,17)} = 13.18$, p = 0.0003; t-test, Tukey correction, wildtype v. heterozygous: $q_{(17)} = 3.693$, p = 0.0457; wildtype v. homozygous: $q_{(17)} = 7.258$, p = 0.0002). **(E)** Neurons were held in current clamp at -70mV. The resonant frequency was measured as the frequency at which the impedance profile reached its peak (arrows). Ratio of the fast Fourier transform of the voltage response (Fig. S6E top) to the fast Fourier transform of the sinusoidal current stimulus (Fig. S6E middle) to calculate the impedance amplitude profile (Fig. S6E bottom). We defined the resonant frequency as the frequency at which the impedance profile reached its peak. Scale bar = 5 mV, 5 s. **(F)** ZD7288, an HCN channel blocker, decreased resonance frequency by over 50% in *Tbr1^{layer5}* heterozygous (green), and *Tbr1^{layer5}* homozygous mutants (blue). **(G)** Quantification of changes in resonant frequency of *Tbr1^{wildtype}* (red), *Tbr1^{layer5}* heterozygous (green) and *Tbr1^{layer5}* homozygous mutants (blue) before and after ZD7288 treatment (n = 7/8/8, wildtype/ heterozygous/ homozygous cells from two different animals/genotype; One-way ANOVA, $F_{(2,20)} = 16.24$, p < 0.0001; t-test, Tukey correction, wildtype v. heterozygous: $q_{(20)} = 7.075$, p = 0.0002; wildtype v. homozygous: $q_{(20)} = 7.038$, p = 0.0002). Grey-filled boxes represent brains that were treated with ZD7288. Floating bar graphs represent the min-max data distribution from each genotype. Horizontal line in each box denotes the average distribution. Average distribution is numerically indicated in each box. (**p< 0.01) (***p<0.001).

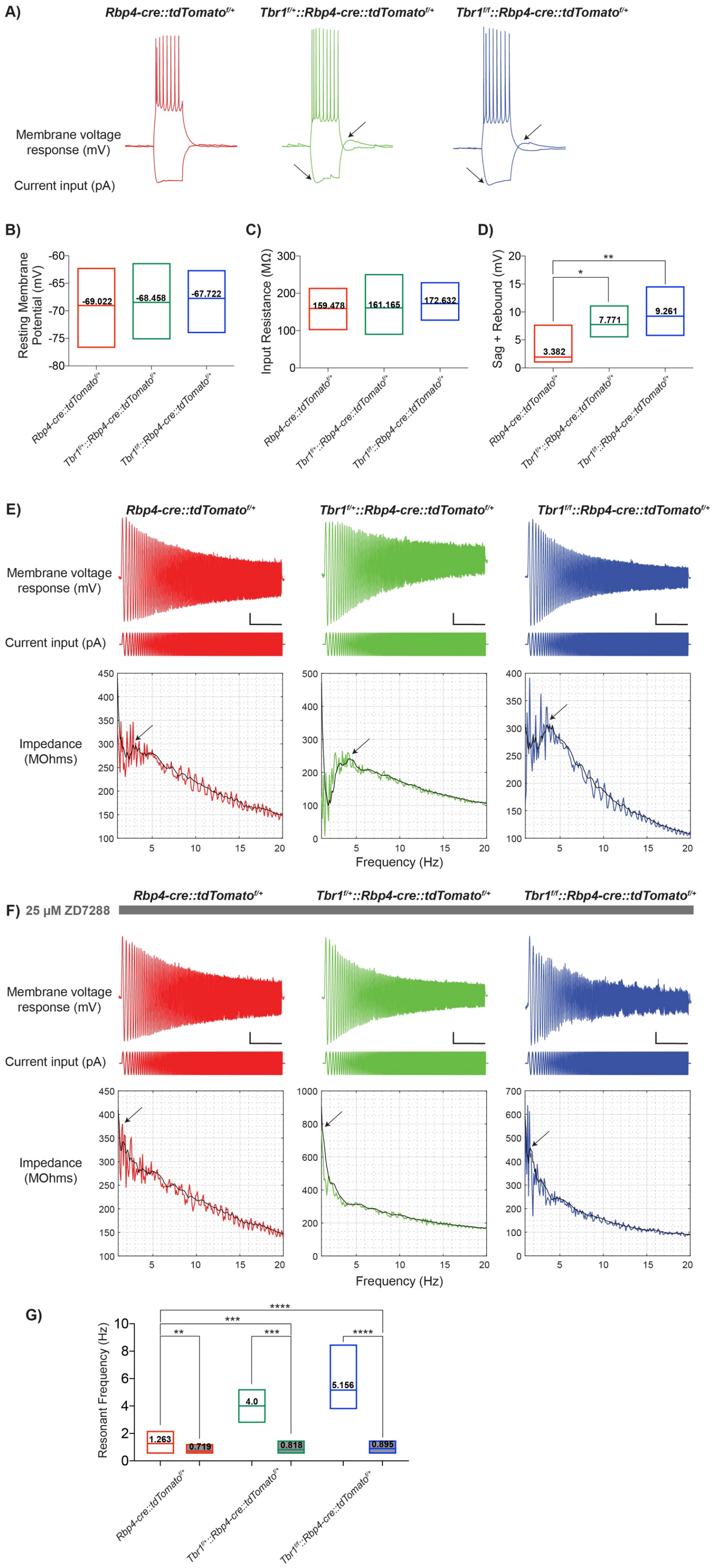

A)
Rbp4-cre::tdTomato f/+
Tbr1 f/+::Rbp4-cre::tdTomato f/+
Tbr1 f/f::Rbp4-cre::tdTomato f/+
Membrane voltage response (mV)
Current input (pA)
B)
Resting Membrane Potential (mV)
-69.022
-68.458
-67.722
C)
Input Resistance (MΩ)
159.478
161.165
172.632
D)
Sag + Rebound (mV)
3.382
7.771
9.261
E)
Impedance (MOhms)
Frequency (Hz)
F) 25 μM ZD7288
G)
Resonant Frequency (Hz)
1.263
0.719
4.0
0.818
5.156
0.895

Figure S6: Related to Figure 4. LiCl rescues dendritic spine density of *Tbr1* mutant neurons. Imaris software was used to quantify dendritic spine density from **(i)** apical dendrites of *Tbr1*^{layer6} CKOs neurons located within layer 5 of SSCx and **(ii)** dendrites of layer 5 neurons from mPFCx of *Tbr1*^{wildtype}, *Tbr1*$^{+/-}$ and layer 6 neurons from SSCx of *Tbr1*^{wildtype} and *Tbr1*$^{+/-}$. Changes in the dendritic spines were examined at P5, P21 (A) and P60 (B-D). Spine density is improved at P5 and P60, 24 hrs after LiCl treatment (A-D), compared to the saline-injected control animals.
(iii) Filamentous spine density is increased in (E) *Tbr1*^{layer5} and (F) *Tbr1*^{layer6} CKO. Layer 5 neurons of *Tbr1*^{wildtype}, *Tbr1*$^{+/-}$ (G) and layer 6 neurons of *Tbr1*^{wildtype} and *Tbr1*$^{+/-}$ (H) showed an increase in filamentous spine density at P60. LiCl treatment of *Tbr1* mutants did not rescue the increase in filamentous spine density (E-H). Floating bar graphs in red (wildtype), green (heterozygotes) and blue (homozygotes) represent the min to max distribution of mature and filamentous spines in *Tbr1* CKOs. Ruby (wildtype) and orange (*Tbr1*$^{+/-}$) represent the distribution of mature and filamentous spines in layer 5 and 6 *Tbr1*$^{+/-}$ neurons. Horizontal line in each box denotes the average distribution. Average distribution is numerically indicated in each box. Two-tailed T-test with Tukey correction was used for pairwise comparisons. ns = not significant. (**$p<0.01$) (***$p<0.001$) (****$p<0.0001$).

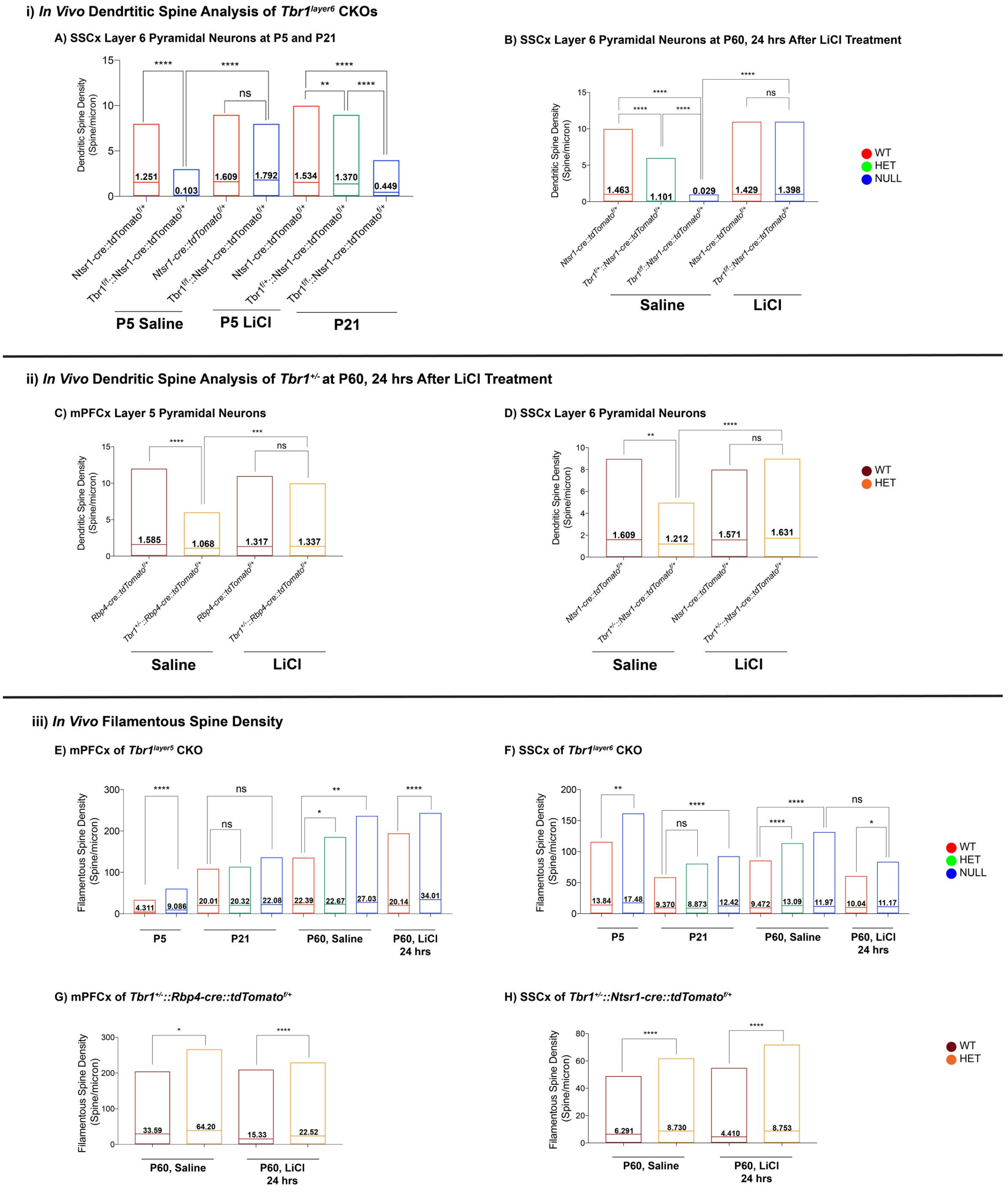
i) In Vivo Dendrtitic Spine Analysis of Tbr1layer6 CKOs
A) SSCx Layer 6 Pyramidal Neurons at P5 and P21
Dendritic Spine Density (Spine/micron)
1.251
0.103
1.609
1.792
1.534
1.370
0.449
Ntsr1-cre::tdTomatof/+
Tbr1f/f::Ntsr1-cre::tdTomatof/+
Ntsr1-cre::tdTomatof/+
Tbr1f/f::Ntsr1-cre::tdTomatof/+
Ntsr1-cre::tdTomatof/+
Tbr1f/+::Ntsr1-cre::tdTomatof/+
Tbr1f/f::Ntsr1-cre::tdTomatof/+
P5 Saline
P5 LiCl
P21
B) SSCx Layer 6 Pyramidal Neurons at P60, 24 hrs After LiCl Treatment
Dendritic Spine Density (Spine/micron)
1.463
1.101
0.029
1.429
1.398
Ntsr1-cre::tdTomatof/+
Tbr1f/+::Ntsr1-cre::tdTomatof/+
Tbr1f/f::Ntsr1-cre::tdTomatof/+
Ntsr1-cre::tdTomatof/+
Tbr1f/f::Ntsr1-cre::tdTomatof/+
Saline
LiCl
WT
HET
NULL
ii) In Vivo Dendritic Spine Analysis of Tbr1+/- at P60, 24 hrs After LiCl Treatment
C) mPFCx Layer 5 Pyramidal Neurons
Dendritic Spine Density (Spine/micron)
1.585
1.068
1.317
1.337
Rbp4-cre::tdTomatof/+
Tbr1+/-::Rbp4-cre::tdTomatof/+
Rbp4-cre::tdTomatof/+
Tbr1+/-::Rbp4-cre::tdTomatof/+
Saline
LiCl
D) SSCx Layer 6 Pyramidal Neurons
Dendritic Spine Density (Spine/micron)
1.609
1.212
1.571
1.631
Ntsr1-cre::tdTomatof/+
Tbr1+/-::Ntsr1-cre::tdTomatof/+
Ntsr1-cre::tdTomatof/+
Tbr1+/-::Ntsr1-cre::tdTomatof/+
Saline
LiCl
WT
HET
iii) In Vivo Filamentous Spine Density
E) mPFCx of Tbr1layer5 CKO
Filamentous Spine Density (Spine/micron)
4.311
9.086
20.01
20.32
22.08
22.39
22.67
27.03
20.14
34.01
P5
P21
P60, Saline
P60, LiCl 24 hrs
F) SSCx of Tbr1layer6 CKO
Filamentous Spine Density (Spine/micron)
13.84
17.48
9.370
8.873
12.42
9.472
13.09
11.97
10.04
11.17
P5
P21
P60, Saline
P60, LiCl 24 hrs
WT
HET
NULL
G) mPFCx of Tbr1+/-::Rbp4-cre::tdTomatof/+
Filamentous Spine Density (Spine/micron)
33.59
64.20
15.33
22.52
P60, Saline
P60, LiCl 24 hrs
H) SSCx of Tbr1+/-::Ntsr1-cre::tdTomatof/+
Filamentous Spine Density (Spine/micron)
6.291
8.730
4.410
8.753
P60, Saline
P60, LiCl 24 hrs
WT
HET

Figure S7: Related to Figure 5. LiCl treatment at P5 and P60 restores normal synapse numbers in *Tbr1* mutant mice, 24hrs after treatment.

Excitatory (i) and inhibitory (ii) synaptic density was quantified at P5 and P60 from (1) apical dendrites of $Tbr1^{layer5CKO}$ and $Tbr1^{layer6CKO}$, 24 hrs after injection with saline or LiCl (n=10 dendrites). Excitatory and Inhibitory synapses were defined by co-localization of $VGLUT1^+$ boutons and $PSD95^+$ clusters (excitatory) and $VGAT^+$ boutons and $Gephyrin^+$ clusters (inhibitory) onto endogenous tdTomato labeling layer 5 and/or layer 6 pyramidal neurons.

(i) Excitatory synapses are quantified from (A) layer 5 neurons of mPFCx of $Tbr1^{wildtype}$ (green) and $Tbr1^{layer5CKO}$ (orange), (B) layer 6 neurons of SSCx of $Tbr1^{wildtype}$ (red) and $Tbr1^{layer6CKO}$ (blue) mice at P60, 24 hrs after saline and/or LiCl was administered.

(ii) Inhibitory synapses are quantified from (E) mPFCx of $Tbr1^{wildtype}$ and $Tbr1^{layer5CKO}$ and (F) SSCx of $Tbr1^{wildtype}$ and $Tbr1^{layer6CKO}$ mice, 24 hrs after saline and/or LiCl was administered at P59. Floating bar graphs represent the min to max distribution of all excitatory and inhibitory synapse numbers measured from each genotype and treatment. Horizontal line in each box denotes the average distribution. Average distribution is numerically indicated in each box. Two-tailed T-test with Tukey correction was used for pairwise comparisons. ns = not significant. (***p<0.001) (****p<0.0001).

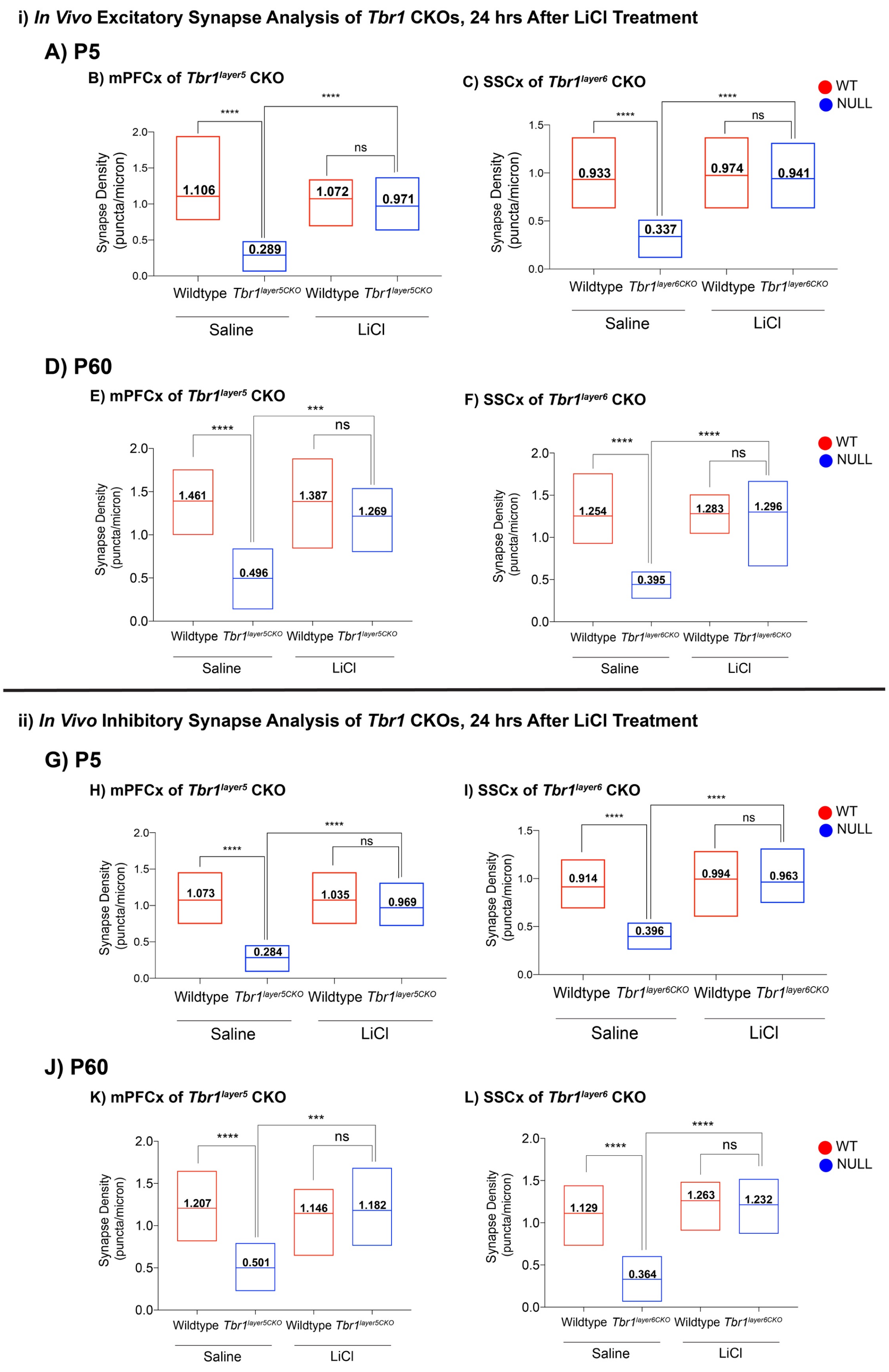
i) In Vivo Excitatory Synapse Analysis of Tbr1 CKOs, 24 hrs After LiCl Treatment
A) P5
B) mPFCx of Tbr1layer5 CKO
Synapse Density (puncta/micron)
1.106
0.289
1.072
0.971
****
****
ns
Wildtype Tbr1layer5CKO Wildtype Tbr1layer5CKO
Saline
LiCl
C) SSCx of Tbr1layer6 CKO
0.933
0.337
0.974
0.941
****
****
ns
Wildtype Tbr1layer6CKO Wildtype Tbr1layer6CKO
Saline
LiCl
WT
NULL
D) P60
E) mPFCx of Tbr1layer5 CKO
1.461
0.496
1.387
1.269
****
***
ns
F) SSCx of Tbr1layer6 CKO
1.254
0.395
1.283
1.296
****
****
ns
ii) In Vivo Inhibitory Synapse Analysis of Tbr1 CKOs, 24 hrs After LiCl Treatment
G) P5
H) mPFCx of Tbr1layer5 CKO
1.073
0.284
1.035
0.969
****
****
ns
I) SSCx of Tbr1layer6 CKO
0.914
0.396
0.994
0.963
****
****
ns
J) P60
K) mPFCx of Tbr1layer5 CKO
1.207
0.501
1.146
1.182
****
***
ns
L) SSCx of Tbr1layer6 CKO
1.129
0.364
1.263
1.232
****
****
ns

Figure S8: Related to Figure 5. GSK3β-inhibitor (GSK3βi) treatment at P60 restores normal dendritic spine density and synapse numbers in *Tbr1* CKOs 24hrs after treatment.
Excitatory (i) and inhibitory (ii) synaptic density was quantified at P60 from (1) apical dendrites of $Tbr1^{layer5CKO}$ and $Tbr1^{layer6CKO}$, 24 hrs after injection with saline or GSK3β-inhibitor at P59 (n=15 dendrites). Excitatory and Inhibitory synapses were defined by co-localization of $VGLUT1^{+}$ boutons and $PSD95^{+}$ clusters (excitatory) and $VGAT^{+}$ boutons and $Gephyrin^{+}$ clusters (inhibitory) onto endogenous tdTomato labeling of layer 5 dendrites (in layer 2/3 of $Tbr1^{layer5CKO}$) or layer 6 dendrites (in layer 5 of $Tbr1^{layer6CKO}$).
(i) Excitatory (A) and inhibitory (B) synapses are quantified from layer 5 neurons of mPFCx of $Tbr1^{wildtype}$ (red) and $Tbr1^{layer5CKO}$ (blue) at P60, 24 hrs after saline or GSK3β-inhibitor was administered. (C) Imaris software was used to quantify the changes in mature dendritic spine density of layer 5 neurons of mPFCx of $Tbr1^{wildtype}$ (red) and $Tbr1^{layer5CKO}$ (blue) at P60.
(ii) Excitatory (A) and inhibitory (B) synapses are quantified from layer 6 neurons of SSCx of $Tbr1^{wildtype}$ (red) and $Tbr1^{layer6CKO}$ (blue) at P60, 24 hrs after saline or GSK3β-inhibitor was administered. (C) Imaris software was used to quantify the changes in mature dendritic spine density of layer 6 neurons of SSCx of $Tbr1^{wildtype}$ (red) and $Tbr1^{layer6CKO}$ (blue). Floating bar graphs represent the min to max distribution of all excitatory and inhibitory synapse numbers measured from each genotype and treatment. Horizontal line in each box denotes the average distribution. Average distribution is numerically indicated in each box. Two-tailed T-test with Tukey correction was used for pairwise comparisons. ns = not significant. (***p<0.001) (****p<0.0001).

i) *In Vivo* Analysis of *Tbr1*[layer5] CKOs in mPFCx at P60, 24 hrs After GSK3β-inhibitor Treatment

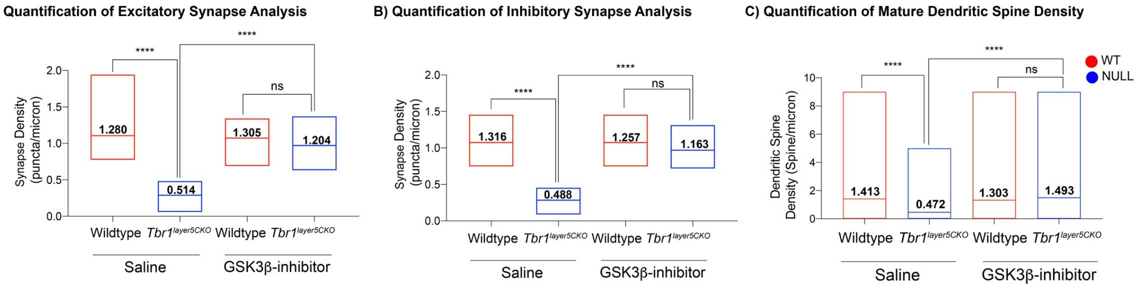

ii) *In Vivo* Analysis of *Tbr1*[layer6] CKOs in SSCx at P60, 24 hrs After GSK3β-inhibitor Treatment

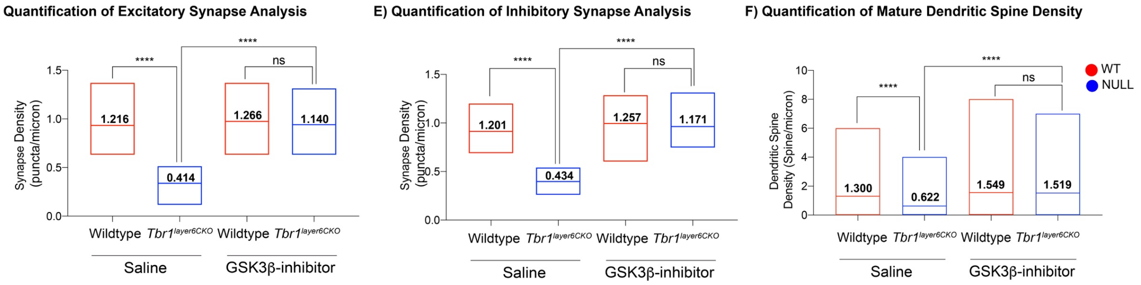

Figure S9: Related to Figure 5. Restoring WNT-signaling rescues synaptic deficit through a cell-autonomous autocrine mechanism.

(i) *In vivo* rescue assay was conducted by injecting a *Cre*-dependent lentivirus expressing *CAG-Flex-IRES-GFP* (empty vector) or *CAG-Flex-Wnt7b-IRES-GFP* lentiviral constructs into layer 6 of SSCx of *Tbr1^{layer6}* wildtype, heterozygous and homozygous CKOs at P1. **(A, B)** Quantification of excitatory and inhibitory synapse numbers onto the layer 6 neurons of *Tbr1^{layer6}* heterozygous (Het-WNT7B-GFP) and homozygous CKOs (Null-WNT7B-GFP) expressing WNT7B-GFP at P21 compared to wildtype (WT-WNT7B-GFP) and empty vector-injected (GFP only) animals including *Tbr1^{layer6}* wildtype (WT; red), heterozygous (Het; green) and homozygous (Null; blue) CKOs. Overexpressing *Wnt7b* in wildtype layer 6 neurons (located by the presence of GFP) did not have an impact on excitatory and inhibitory synaptic density **(A, B)**. However, the regions expressing GFP in layer 6 cells of *Tbr1^{layer6}* CKOs, showed an increase in excitatory and inhibitory synapse numbers **(A, B)**.

(ii) Transplant-mediated introduction of cortical interneurons expressing *Wnt7b* to test whether *Wnt7b* promotes synaptogenesis through a paracrine mechanism. Immature cortical interneurons (MGE donor cells; *Nkx2.1-cre::tdTomato$^{f/+}$)* were transfected with lentiviral constructs encoding *Gfp* [*DlxI12b-GFP* (control)]or encoding *Wnt7b* and *Gfp* (*DlxI12b-Wnt7b-GFP*). Transfected cells were transplanted in the P1 neocortex *Tbr1^{layer6}* wildtype (WT; red) and homozygous CKO (Null; blue) and analyzed at P30. *Ntsr1-cre::tdTomato$^{f/+}$* allele was used to label the layer 6 neurons (red). **(C)** 4X and 10X magnification of tdTomato signal from transplanted interneurons within layer 5 of SSCx, and apical dendrites of layer 6 pyramidal neurons. MGE transplanted cells were identified as being both tdTomato^{+} and GFP^{+}. **(D)** ImageJ software was used to process confocal images for quantification. **(E)** Quantification of excitatory synaptic density onto layer 6 dendrites of *Tbr1^{layer6}* wildtype (WT; red) and homozygous CKO (Null; blue) within layer 5 of SSCx at P30. **(F)** Quantification of excitatory synapses onto the soma of transplanted interneurons expressing either an empty vector control (*DlxI12b-GFP*) or the *Wnt7b* and *Gfp* vector (*DlxI12b-Wnt7b-GFP*).

(iii) *In vitro* rescue assay was conducted using *Cyp26b1* expression vector in cultured P0 neocortex from *Tbr1^{wildtype}* (red) and *Tbr1^{layer6}* CKOs (blue) (n=2). Excitatory and Inhibitory synapses were measured at P14. Synapses are defined by co-localization of VGLUT1^{+} boutons and PSD95^{+} clusters (excitatory) and VGAT^{+} boutons and Gephyrin^{+} clusters (inhibitory) onto endogenous tdTomato. **(G, H)** Quantification of excitatory and inhibitory synaptic density *in vitro*. Restoring *Cyp26b1* expression *in vitro* rescues excitatory and inhibitory synaptic deficit in *Tbr1^{layer6}* CKOs (red) compared to wildtype control (blue). Floating bar graphs represent the min to max distribution of synaptic density measured from all genotypes and treatments. Horizontal line in each box denotes the average distribution. Average distribution is numerically indicated in each box. Two-tailed T-test with Tukey correction was used for pairwise comparisons. ns = not significant. (**p< 0.01) (***p<0.001) (****p<0.0001).

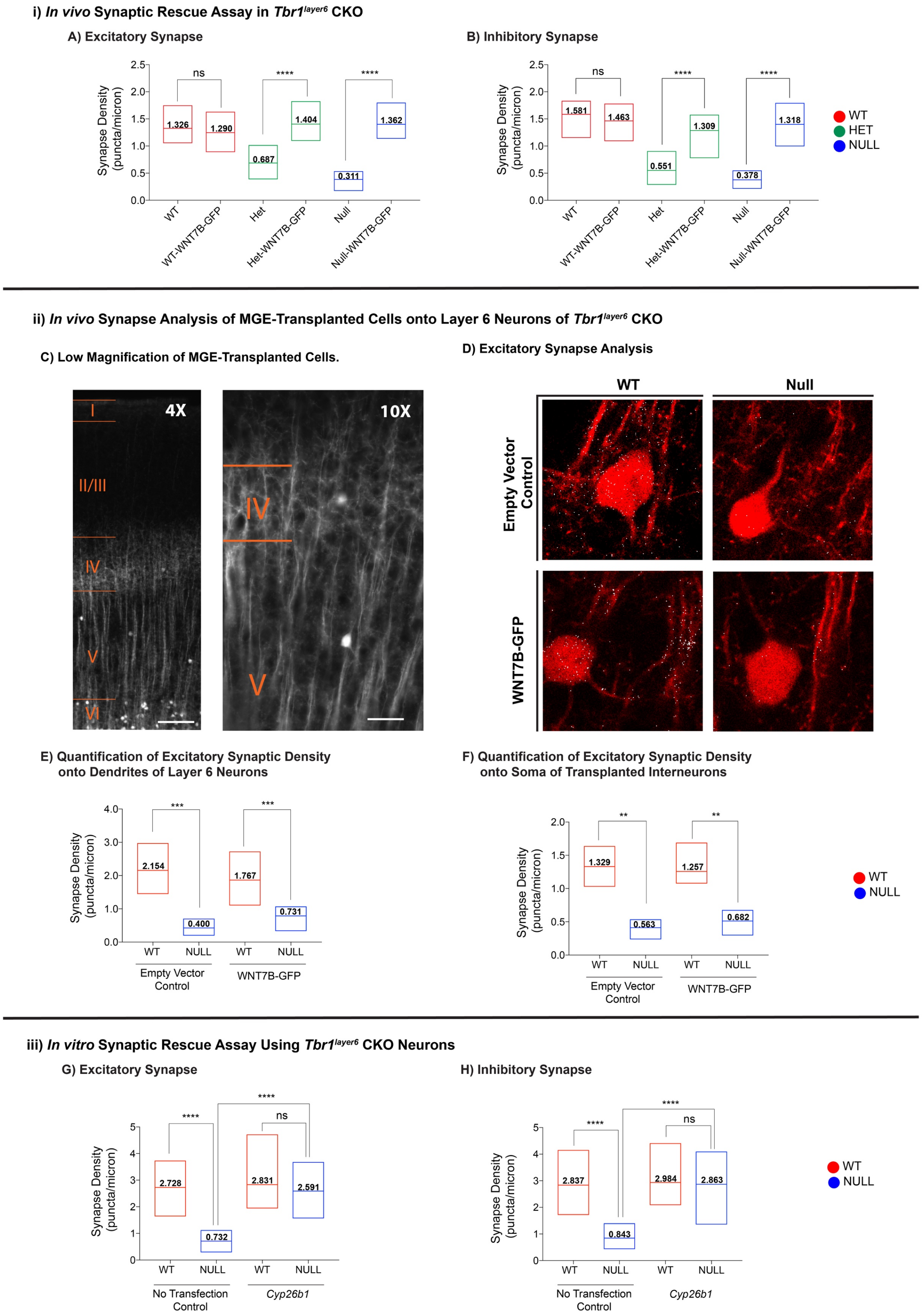
i) In vivo Synaptic Rescue Assay in Tbr1layer6 CKO
A) Excitatory Synapse
B) Inhibitory Synapse
Synapse Density (puncta/micron)
ns
****
1.326
1.290
0.687
1.404
0.311
1.362
1.581
1.463
0.551
1.309
0.378
1.318
WT
WT-WNT7B-GFP
Het
Het-WNT7B-GFP
Null
Null-WNT7B-GFP
WT
HET
NULL
ii) In vivo Synapse Analysis of MGE-Transplanted Cells onto Layer 6 Neurons of Tbr1layer6 CKO
C) Low Magnification of MGE-Transplanted Cells.
4X
10X
I
II/III
IV
V
VI
D) Excitatory Synapse Analysis
WT
Null
Empty Vector Control
WNT7B-GFP
E) Quantification of Excitatory Synaptic Density onto Dendrites of Layer 6 Neurons
***
2.154
0.400
1.767
0.731
F) Quantification of Excitatory Synaptic Density onto Soma of Transplanted Interneurons
**
1.329
0.563
1.257
0.682
WT
NULL
Empty Vector Control
WNT7B-GFP
iii) In vitro Synaptic Rescue Assay Using Tbr1layer6 CKO Neurons
G) Excitatory Synapse
H) Inhibitory Synapse
2.728
0.732
2.831
2.591
2.837
0.843
2.984
2.863
No Transfection Control
Cyp26b1
